# Global systematic review with meta-analysis reveals yield advantage of legume-based rotations and its drivers

**DOI:** 10.1038/s41467-022-32464-0

**Published:** 2022-08-22

**Authors:** Jie Zhao, Ji Chen, Damien Beillouin, Hans Lambers, Yadong Yang, Pete Smith, Zhaohai Zeng, Jørgen E. Olesen, Huadong Zang

**Affiliations:** 1 College of Agronomy and Biotechnology, China Agricultural University, Beijing, China; 2 Department of Agroecology, Aarhus University, Blichers Allé 20, 8830 Tjele, Denmark; 3 CIRAD, UPR HORTSYS, Montpellier, France; 4 School of Biological Sciences and Institute of Agriculture, The University of Western Australia, 35 Stirling Highway, Crawley (Perth), WA 6009 Australia; 5 Institute of Biological and Environmental Sciences, University of Aberdeen, 23 St Machar Drive, AB24 3UU Aberdeen, UK

**Keywords:** Agroecology, Biodiversity, Biodiversity

## Abstract

Diversified cropping systems, especially those including legumes, have been proposed to enhance food production with reduced inputs and environmental impacts. However, the impact of legume pre-crops on main crop yield and its drivers has never been systematically investigated in a global context. Here, we synthesize 11,768 yield observations from 462 field experiments comparing legume-based and non-legume cropping systems and show that legumes enhanced main crop yield by 20%. These yield advantages decline with increasing N fertilizer rates and crop diversity of the main cropping system. The yield benefits are consistent among main crops (e.g., rice, wheat, maize) and evident across pedo-climatic regions. Moreover, greater yield advantages (32% vs. 7%) are observed in low- vs. high-yielding environments, suggesting legumes increase crop production with low inputs (e.g., in Africa or organic agriculture). In conclusion, our study suggests that legume-based rotations offer a critical pathway for enhancing global crop production, especially when integrated into low-input and low-diversity agricultural systems.

## Abstract (web summary)

Crop rotations including legumes have been proposed as a strategy to enhance food production. Here, the authors conduct a global meta-analysis on legume-based crop rotations, showing that legume pre-crops increase 20% of yield in average across various crops and climatic regions.

## Introduction

Enhancing biodiversity spatially (e.g., intercropping) or temporally (e.g., crop rotation) in cropping systems may promote ecosystem services, such as pest and disease control^1^, carbon sequestration, and soil fertility^2,3^. These services potentially reduce the dependence on external inputs while maintaining high crop yields and production stability^1,4^. Diversification through the inclusion of legumes in cereal-, root-, or tuber-based cropping systems represents a key strategy for sustainable agriculture^3,5,6^. However, legume cultivation has declined globally in recent decades due to their low and unstable yields, leading to reduced high-quality protein production and loss of ecosystem services^7,8^. The legumes species (i.e., legumes grown for grain, forage, and green manure) affect its biomass production and the quantity and quality of crop residues, which further influence the potential benefits for subsequent crops in the rotation^9^. Therefore, designing and (re)building legume-based cropping systems will likely enhance local and global crop production while minimizing negative environmental impacts.

Recent quantitative syntheses of legume pre-crop effects on following crop yield have either been limited to specific crop species^10^ or confined to regional scales^11–14^. Additional studies are needed to determine the combined effects of environmental and agronomic factors (e.g., initial crop diversification and N fertilization) on the main crop yield after legume inclusion. A global-scale quantitative synthesis, over a broad range of environmental conditions, could identify the magnitude and variability of legume pre-crop effects in diversified cropping systems, responding to the growing interest in legume and crop diversification among the agricultural research community, farmers, and value chain actors^2,13,15^. The crop yield after legumes is often enhanced due to the combined and interrelated effects of nitrogen (N) provision and non-N effects (e.g., suppressed pest and disease, improved soil properties)^2,16,17^. Therefore, our first hypothesis is that N fertilization for main crops will reduce the legume pre-crop effect due to suppressed nodulation and N_2_ fixation^16–18^. Since rotation with non-legumes can also enhance the yield of following crops due to break-crop (non-N) effects^10,11^, our second hypothesis is that crop diversification, in terms of crop species diversity, functional diversity, and the number of crops per year (temporal diversity), will decrease the (non-N) legume pre-crop effect. In addition, our understanding of the key environmental and management factors determining the main crop yield after legume inclusion still remains incomplete. Therefore, a comprehensive quantitative synthesis at a global scale is crucial to help make informed decisions about how legume-based rotations can be designed and implemented better.

**Fig. 1:**
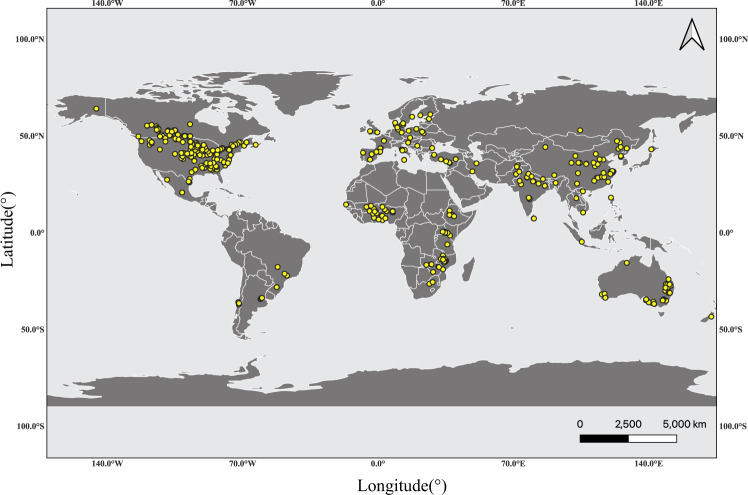
Global distribution of rotation experiments testing the effects of legume pre-crop on main crop yield included in our meta-analysis. The data set covers 11,768 paired yield observations from 462 field experiments across 53 countries from 1959 to 2020. The map was created with QGIS version 3.20 (Open Source Geospatial Foundation Project, http://qgis.osgeo.org). Global map was downloaded from natural earth (http://www.naturalearthdata.com/).

We performed a systematic review with meta-analysis on data from peer-reviewed publications related to the yield advantages of legume-based rotations in 462 studies with 11,768 paired observations across 53 countries (Fig. 1). Only field experiments containing side-by-side yield comparisons were included in the database (see “Methods” for study selection details). Our analysis allows precise quantification of the change in the subsequent crop yield after legume inclusion for various cropping systems depending on initial crop diversification, N fertilization, or crop types.

## Results and discussion

### Legume pre-crop effect at a global scale

**Fig. 2:**
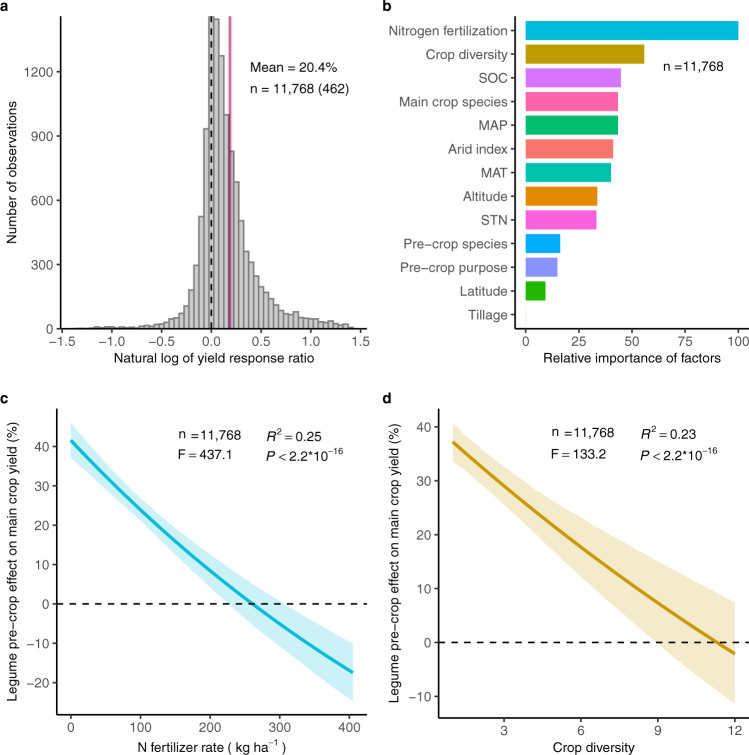
Overall effect, variable importance, and the effect of two most important factors determining the legume pre-crop effect on main crop yield. **a** Histogram of natural log response ratios (ln*RR*s). **b** Importance of factors in determining the yield benefit of the legume-based cropping system. **c** The yield effect of legume pre-crop decreased significantly with increasing N fertilizer rate. **d** The yield effect of legume pre-crop decreased significantly with increasing crop diversity. Crop diversity is defined as the number of crop species × number of crop functional groups × number of crop species per year. The dashed line, solid red line, and the shaded area in a represent ln*RR* = 0, the mean, and the standard errors of the mean on a natural log scale. The numbers denote the total observations with the number of studies in parentheses. The relative importance in **b** is quantified based on a meta-forest model. MAT, MAP, SOC, and STN stands for mean annual temperature, mean annual precipitation, initial soil organic carbon and total nitrogen content in topsoil, respectively. Linear mixed-effects model fit tests used Satterthwaite approximations for denominator degrees of freedom. The effects are quantified as the percentage changes in legume- versus non-legume-based cropping systems. Colored lines in **c**, **d** represent the average N fertilizer rate- and diversity-specific responses, respectively, with their bootstrapped 95% confidence intervals indicated by shading.

Our meta-analysis contains 60 major legume-based cropping systems from field studies and covers 53 countries from 1959 to 2020. The results show that among the main crops following legumes, 91% involved cereals, 6% were rapeseed, 2% were roots and tubers, and <1% were fodder, fiber, and sugar crops. For crop diversity of the initial cropping systems (i.e., the control), 45% of observations were obtained in monoculture (crop diversity  = 1) and 47% in short rotations (crop diversity ≤ 4). This large and detailed dataset provides the basis for profound understanding of the legume pre-crop effects, which cannot be drawn from earlier regional meta-analyses^11–14^. We found that the legume pre-crop effect on main crop yield was predominantly positive (73.6% positive, 0.7% neutral, and 25.7% negative), with 50% of the data showing a yield increase >10% (Fig. 2a). Averaged across all studies, legumes significantly increased subsequent crop yield by 20.4% (median effect, 10.2%; 95% confidence intervals (CIs), 17.7–23.1%; *P* < 0.001, Fig. 2a). This yield benefit was less than the estimate of 29%, which was synthesized from 1181 observations for grain legume pre-crop effect on cereals at a global scale^16^. Our study yet confirms an important effect of the legumes advantage on a wider range of crops by including additional legumes used for fodder and green manure, and additional main crop categories such as oilseed, fiber, root, and tuber crops. Thus, our results provide detailed supporting evidence that main crops have, in average, a higher yield following legume than after non-legume pre-crops at the global scale.

### Crop diversity and N fertilization of the initial cropping system mediate legume pre-crop effect

Growing crops in monoculture or short rotations (i.e., low crop diversity) has become more prevalent worldwide due to economic, technological, and policy influences^19^. By using a machine learning algorithm (a random-forest approach) in the context of a meta-analysis (meta-forest) (See “Methods”), we found that, over a broad range of pedo-climatic and management factors, the legume pre-crop effect was predominantly explained by crop diversity of the initial cropping system (defined as the number of crop species × number of crop functional groups × number of crop species per year) and N fertilizer rate (Fig. 2b). Moreover, these two factors were always the most important moderators in sensitivity analyses performed by removing the variables containing the most frequently missing values (Supplementary Fig. 1). The legume pre-crop effect was negatively correlated with the N fertilizer rate of the main crop, as the yield advantage decreased by 7% for each 50 kg N ha^–1^ added (Fig. 2c). This effect was consistent among main crops, including major (wheat, rice, and maize) and secondary cereals (except millet), rapeseed, cotton, and potato (Supplementary Fig. 2). Similarly, increasing crop diversity by 1-unit led to a 3.5% decrease in yield advantages of legume pre-crops (Fig. 2d). This means, for example, that the addition of one species (or functional group) in the cropping sequence will reduce the main crop yield gain from a previous legume by 3.5% on average. Yet, large uncertainty remains for high-diversity cropping sequences, as shown by the wide CIs. This reduced yield advantage with increased initial crop diversity was significant for wheat, maize, rice, barley, and rapeseed after legume pre-crop, but not significant for sorghum, millet, cotton, and potato (Supplementary Fig. 3). For example, with 1-unit increase in the crop diversity, the legume pre-crop effect on main crop yield decreased on average by 2.9% and 3.6% for wheat and barley, respectively. These findings extend our understanding of the critical role of diversified cropping beyond the benefits of legumes in crop production.

**Fig. 3:**
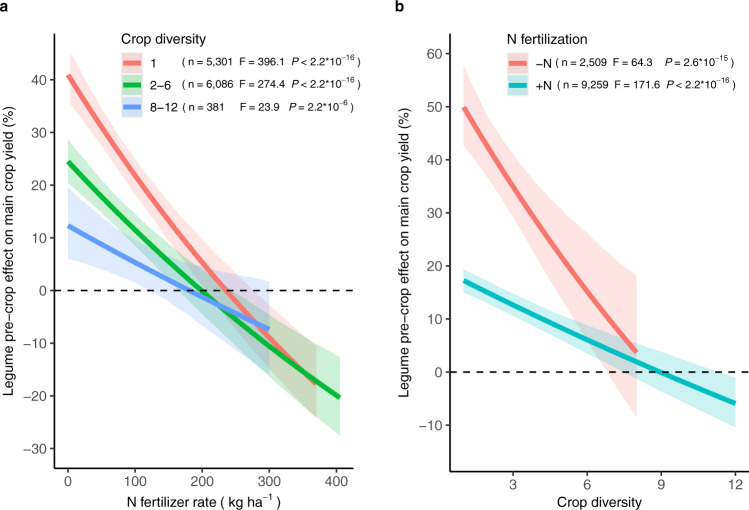
Comparison of main crop yield in legume- versus non-legume-based cropping system. **a** The correlation between the legume pre-crop effect on main crop yield and the N fertilizer rate depending on crop diversity. **b** The correlation between the legume pre-crop effect on main crop yield and crop diversity depending on N fertilization (−N, without N fertilizer; +N, with N fertilizer). Colored lines represent the average diversity- or fertilization-specific responses, with their bootstrapped 95% confidence intervals indicated by shading. Crop diversity is defined as the number of crop species × number of crop functional groups × number of crop species per year. Linear mixed-effects model fit tests used Satterthwaite approximations for denominator degrees of freedom. The significance (*P*) is presented for each term tested.

For the legume pre-crop effect, we found a significant interaction between N fertilizer rate and crop diversity (*P* < 0.001, Fig. 3). The inclusion of legumes in monoculture (crop diversity = 1) results in high yield advantages in the unfertilized condition (+41.0%), but a rapid decrease of the effect with fertilization (–7.9% for each 50 kg N ha^–1^ application) (Fig. 3a). In contrast, with a high level of crop diversification, the average effect of legumes is lower in the unfertilized condition (+12%), but decreases slowly with fertilization (–3.2% for each 50 kg N ha^–1^ application). The positive effect of legumes on main crop yield becomes null with N fertilizer rates higher than 236 kg N ha^–1^ regardless of crop diversification. The yield advantage provided by legumes is strong (+50%) at low crop diversity (crop diversity = 1) and decreases sharply with increased crop diversification (–6.6% for each unit of diversity) in the unfertilized condition (Fig. 3b). In contrast, with N fertilization, the average effect of legumes is lower at low crop diversity (+17%), and decreases more slowly with increased crop diversification (–2.1% for each unit of diversity). The effect of legumes inclusion was no longer significant for highly diversified cropping system (i.e., crop diversity > 9.0), regardless of N fertilization. Our results quantified the yield advantages of legume inclusion at global scale and assessed how this was affected by N fertilization and crop diversity of the initial cropping system.

The legume pre-crop effect is predominantly associated with the N benefit from legume residues and biological N fixation (BNF)^18,20,21^. We identified that the legume pre-crop effect was best predicted by the N fertilizer rate, indicating that judicious use of N fertilizer is critical for the synergistic benefits in crop rotations. Two possible causes may account for the negative response of the legume pre-crop effect to N fertilization. First, the higher N fertilizer rate will meet the crop demand to a greater extent, largely offsetting the N benefits from the legume pre-crop. Second, long-term legacy effects of N inputs will maintain a high level of soil available N, which suppresses the legume BNF and decreases the carryover effect of ‘spared’ mineral N^17,18,22^. This explanation is supported by the fact that the cover and biomass of grassland legumes declined substantially with increasing N additions^23^. Remarkably, the slope of the legume pre-crop effect versus N fertilizer rate decreased with crop diversity, indicating that the yield benefit was less susceptible to the N application with more complex cropping sequences^19^. Therefore, our results strongly suggest that the legume pre-crop effect was mainly accounted for by the N benefit and this can be replaced partly by increased N fertilization.

More importantly, the strong negative relationship between crop diversity and the legume pre-crop effect indicates that the yield benefits from legume inclusion were more important for the cropping system with lower diversity. Crop diversification of the non-legume cropping system (i.e., the control) enhanced main crop yield via the inherent capacity of the break-crop (non-N) effect, thus partly closing the yield benefit gap between non-legume and legume pre-crops^10,11^. This break-crop effect strongly relies on the reduced abiotic (e.g., drought and nutrients) and biotic (e.g., pests, weeds, and diseases) stressors through improvement in soil biological, biochemical, and structural properties in the non-legume cropping system^10,14,24^. These findings indicate that cropping sequences can be diversified without legumes to achieve potential yield advantage, where legume pre-crops are not agronomically or economically viable^13^. Furthermore, legumes maintained a certain yield advantage, even under relatively high crop diversity, which provides broad support for the hypothesized link between the decline of legumes in cereal rotations and stagnation of wheat yields in Europe^25^. Our results highlight that food production could be boosted through higher crop diversity (especially legume-based systems) and optimized N fertilization.

### Greater legume pre-crop effect at low yield levels and low N inputs

**Fig. 4:**
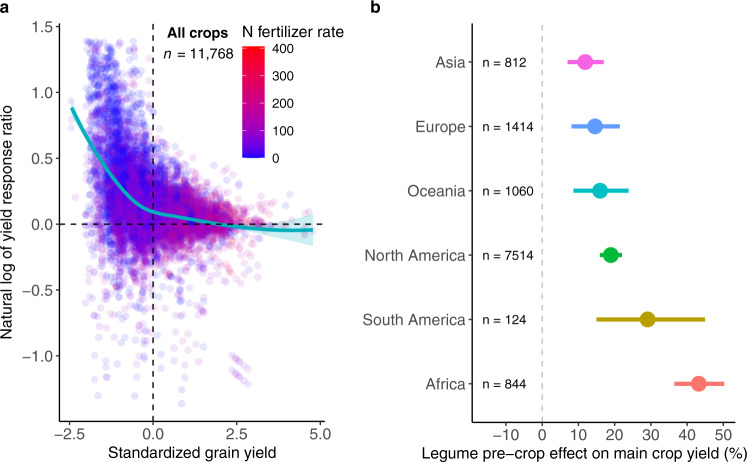
Legume pre-crop effect on main crop yield as a function of yield level and among global regions. **a** The log yield response ratio (yield of crops following legumes vs. non-legumes) decreased with increasing yield level with a more pronounced pre-crop effect at low N fertilizer rate. **b** Legume pre-crop effect on main crop yield across global regions. The dashed horizontal and vertical lines in **a** were drawn at ln*RR* = 0 and averaged grain yield (standardized grain yield = 0), respectively. Standardized grain yields were calculated by observed values minus mean and divided by one standard deviation. The solid line and shading area in **a** represent the smoothed conditional average response and the 95% confidence interval (CI) fitted by local weighted regression using “loess” function in R. Dots and error bars in **b** represent overall effect sizes from a meta-analysis and 95% CIs. For each region, the numbers denote the total observations with the number of studies in parentheses. Yield benefits differ significantly among global regions, as indicated by non-overlapping CIs.

Greater legume pre-crop effects were observed at low yield levels and low N inputs (Fig. 4). For all main crop species, the yield advantages after legume inclusion declined sharply with increasing initial main crop yield. For the main crop yield above the average yield observed in our database, the yield advantage was nearly constant and negligible after legume inclusion (Fig. 4a). Moreover, 79% of the observed greater yield increment (>20%) was found at initial yields lower than the average level. Consistent with the overall declining trend of all main crop species, the pre-crop effect on major (wheat, maize, and rice) and secondary (barley) cereals decreased sharply until the yield reached the average level (Supplementary Fig. 4). The legume pre-crop effect became negligible beyond the average yield, suggesting that legume inclusion has limited benefits for the high-yielding cropping system.

A greater legume pre-crop effect at low yield levels may partially be attributed to the recovery from stress conditions of the main crop such as N deficiency and other biotic and abiotic stresses^26^. These results substantiate the critical role of legumes in low-input and low-yielding farming systems. For example, in Africa, crop yields are much lower than the attainable yield, mainly due to land degradation and low N fertilizer inputs^27^. Our results confirm that the introduction of legumes into such cropping systems improved the main crop yield significantly (43%), more that twice that in North America (19%), and more than three times that in Asia (12%) (Fig. 4b). Another important application occurs in organic agriculture, where yields are often lower than in conventional high-input agriculture^28^. Conversion of current cropland to organic farming requires inclusion of legumes to improve N availability when there is limited input of organic or mineral fertilizer^29–32^. Thus, our results show that the inclusion of legumes enhances subsequent crop yield, especially in farming systems with low yields and low N fertilizer input.

### Cropping sequences and management affects the legume pre-crop effect

**Fig. 5:**
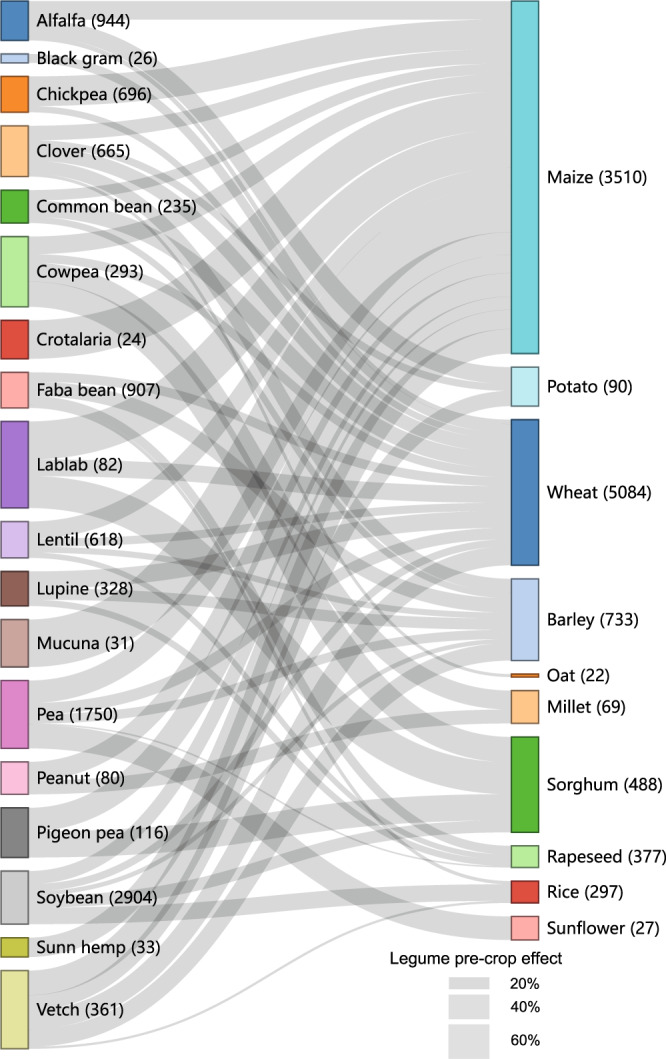
Associations between legume pre-crops and their rotation effects on yield of main crops. The specific flow width represents the magnitude of legume pre-crop effect on main crop yield. Here, cropping sequences are shown when the number of observations (the numbers in parentheses) is more than 20.

Optimizing cropping sequences that maximize positive interspecific plant-soil feedback might improve the efficiency of crop rotation schemes^33,34^. We found that cropping sequences significantly affected the yield advantage of 60 major legume-based cropping systems, with a minimum and maximum value of 2% (CI, –2.9%–7.7%) and 78% (CI, 59.1%–99.4%) in pea-rapeseed and mucuna-maize cropping sequences, respectively (Fig. 5). Consistent with previous reviews on rotation effects^16,26^, the variable legume pre-crop effect indicates that beneficial crop interactions are specific among cropping sequences. The legume-cereal sequence had a significant yield advantage of 21% (CI, 18.8%–23.7%) in contrast to non-legume-cereal sequence (Supplementary Fig. 5). The legume pre-crop effect varied with legume and main crop species, and was not always statistically significant (Supplementary Tables 1 and 2), which may be explained partly by their capacity for BNF^35,36^ and crop-specific traits^37–39^. For example, pigeon pea increased main crop yield by 32.4% (CI, 23.8%–41.0%), more than twice as much as common bean (14.5%; CI, 9.5%–19.5%). Legume inclusion had a significant yield benefit for the following maize (28.9%; CI, 25.5%–32.2%), while the benefits are null for the following rapeseed (3.9%; CI, 0.0%–7.8%), cotton (–1.3%; CI, –12.0%–9.5%), and buckwheat (5.4%; CI, –3.5%–14.2%). Moreover, other management practices such as legume purpose, tillage, and soil parameters (e.g., soil carbon content and texture) also affected the legume pre-crop effect (Supplementary Fig. 6). Thus, integrated management by considering conservation tillage and straw mulching is needed to further improve the sustainability of legume-based cropping systems^40,41^.

In summary, we conducted a global meta-analysis of the impacts of legume-based rotations on main crop production. Legume pre-crops increased the yield of the main crop, on average by 20% globally, with significant variation among continents, ranging from 12% in Asia to 43% in Africa. N fertilization and crop diversity exerted greater control over crop yield benefits from including legumes in rotation than a broad suite of climatic and edaphic factors, and this was the case across various crop species. The positive yield effects of legume pre-crops decreased with increasing N fertilization and crop diversity of the initial cropping system. This implies that yield benefits can be achieved through optimizing the legume pre-crop effect with reduced N fertilization, or through increased crop diversity even without legumes. Legumes have a stronger influence on the main crop when their initial yield was below average. Overall, our results illustrate the critical roles of legumes in yield improvement in low-input and low-diversity farming systems, while also suggesting the potential for reducing N inputs in intensive high-yield cropping systems. It should be noted that growing legumes may reduce the area of the main crop, which might compromise global food security in the short term. However, this negative effect can be partly mitigated by the yield benefits after legume inclusion. Moreover, food security emphasizes not only quantity but also quality and sustainablity. Legume-based cropping systems provide high-quality proteins, which are important for a healthy diet. Therefore, legume-based rotations provide a critical opportunity for enhancing global crop production and advancing the sustainable intensification of agriculture.

## Methods

### Data collection

#### Literature search

We comprehensively searched Web of Science (http://www.webofknowledge.com), Google Scholar (The first 5000 records, http://scholar.google.com), and the China National Knowledge Infrastructure (CNKI; https://www.cnki.net) for studies that examined the yield effect of legume-based cropping systems. The last search was done in October 2020 with the equation: ("crop* rotation*" OR "crop* sequence*" OR "sequential crop*" OR "successive crop*" OR "ley farming" OR "sequence* of plant species" OR "sequence* of crops") AND ("yield*") adapted from Hufnagel et al. (2020)^42^. We also checked the reference list within the selected papers after screening, the previous meta-analyses^10–14,16,24,43–45^, and reviews^19,26,46–52^ on the subject. The literature search was based on the procedure of PRISMA (Preferred Reporting Items for Systematic Reviews and Meta-Analyses)^53^ (Supplementary Fig. 7).

#### Study selection

Publications were screened based on the following criteria: (1) the rotation experiment was conducted under field conditions and contained side-by-side comparisons of legume and non-legume pre-crop rotations with the same main crop; (2) subsequent crop yield data were reported or could be calculated; (3) the initial climatic conditions, soil properties, and main crop management practices were the same; (4) location of the experiment was stated. In addition, we considered different experiments to be distinct studies when multiple experiments (usually locations) were reported in one publication. When multiple publications originated from the same experiment (usually a long-term experiment), we coded them as the same study. If a publication reported different ‘rotation cycles’ of the crop rotation, we included each cycle independently. When different publications included the same data, we recorded the data only once. In total, 462 studies reported in 476 papers published between 1959 and 2020 were included in this meta-analysis (Supplementary note).

#### Data extraction

For each study, we extracted the means, the number of replications, and indicators of precision of the effect-sizes (standard error or confidence intervals). Grain yield and moisture recorded from each crop were converted to kilograms per hectare and adjusted to moisture contents of 15.5% (maize), 15% (barley), 14% (wheat, rice, and millet), 12% (sorghum and rapeseed). We standardized the yield for all main crops in the non-legume cropping systems (observed values minus mean and divided by one standard deviation) for further analysis. In addition to the response variable, our database also included site characteristics like location, climate, and soil quality, and management information like crop species and cultivation practices, which we used to explain the variation in ln*RR* (see Supplementary Table 3). In cases where data were only presented in figures, values were extracted using the GetData Graph Digitizer (http://getdatagraphdigitizer.com/). In a few instances where yield data were only reported as a percentage change relative to other treatments, we assumed absolute yield values for the reference treatment and calculated the natural log of the response ratio^54^.

The digital latitude and longitude of each site were recorded or derived by the location of the nearest city or the experimental station at which the study took place (Supplementary dataset 1). Topographical conditions, i.e., altitude, climate variables including mean annual temperature (MAT), mean annual precipitation (MAP), and annual aridity index (AI, mean annual precipitation divided by potential evapotranspiration) were collected from studies or extracted from the WorldClim database^55^ using site geographic location (i.e., latitude and longitude). Soil physicochemical properties including initial pH in water, soil organic carbon (SOC), and total N concent (STN) were collected from studies or extracted from the HWSD database^56^ using latitude and longitude coordinates. Soil texture was classified into 11 textural classes and further grouped into three categories based on the USDA textural classes of soils^57^. In a few cases (<1%) where only large geographical areas were stated in publications, climate and soil variables were not estimated.

A major objective of our study was to test the rotation effect of legume pre-crops on non-legume main crop yield. To do this, we identified features for both (control and treatment) cropping systems, including preceding legume crop species, legume crop purpose, subsequent crop species, crop types, number of crops per year, and crop diversity. Preceding legume crop species were extracted from the studies and classified to a unique common name based on their scientific name. Legumes were classified as grain legumes, fodder legumes, and green manure legumes, based on their purpose. Subsequent crop species were classified as crop functional groups based on FAO definitions^58^. Crop diversity was calculated for each non-legume cropping system (i.e., the control) by Eq. (1) to simultaneously capture differences in crop species and functional diversity on spatial and temporal scale^4,59,60^

1 $${{{{{\rm{Crop}}}}}}\,{{{{{\rm{diversity}}}}}}={N}_{{{{{\rm{species}}}}}}\times {N}_{{{{{\rm{group}}}}}}\times {N}_{{{{{\rm{year}}}}}}$$

where *N*_species_ is the total number of crop species, *N*_group_ is the total number of crop functional groups, and *N*_year_ is the average number of crop species per year. We calculated an index of crop diversity based on the number and type of cultivated crop species and also considered the cropping intensity of the non-legume cropping system (i.e., the control). Crop species are the base of the crop diversity, which directly affects the diversity of the crop rotation. The crop functional group is considered to disentangle the diversity of the species with similar responses to the environment and with similar effects on ecosystem functioning. For instance, oat and wheat belong to the same functional group, while oat and sunflower belong to the different functional groups. The number of crop species per year is used to distinguish cropping intensity (e.g., two harvests vs. one harvest per year). Detailed examples for crop diversity calculation are shown in Supplementary Table 4.

Management practices including residue management, conservation tillage, irrigation practices, N fertilizer rate, and rotation cycle were recorded for each study as categorical or continuous variables where possible. Residue management was treated as a binary variable (retained/removed), where ‘retained’ indicates that crop residues were retained in the field following harvest, and ‘removed’ indicates that residues were physically removed from the field or burned following harvest. Conservation tillage was also treated as a binary variable (yes/no), where ‘yes’ indicated that the main crop was tilled by conservation tillage, including no-till, strip-till, ridge-till, and ‘no’ indicated conventional tillage, including mold-board and chisel plow, applied to the main crop. Irrigation practices (yes/no) were recorded when available, with cells left blank when irrigation practices were unclear. For multi-year rotation experiments, we used the rotation cycle to quantify the rotation effect over time^61^. Information for categorical variables was extracted from the Materials and Methods section of publications, and to a lesser extent was inferred from discussions of crop management details found in the Introduction or Discussion sections of the papers. The full dataset generated in this study are available in Zhao et al.^62^.

### Data analysis

#### Effect size calculation

The natural log of the response ratio (ln*RR*) was calculated to measure the effect of legume-based rotations on subsequent crop yields following Hedges et al. (1999)^63^:

2 $${{{{{\mathrm{ln}}}}}}\,RR=\,{{{{{\mathrm{ln}}}}}}({\bar{X}}_{t}/{\bar{X}}_{c})=\,{{{{{\mathrm{ln}}}}}}\,{\bar{X}}_{t}-\,{{{{{\mathrm{ln}}}}}}\,{\bar{X}}_{c}$$

where ${\bar{X}}_{t}$ and ${\bar{X}}_{c}$ are the mean yield of the subsequent crop in the legume- and non-legume-based cropping system, respectively. Because standard deviations or the standard errors were available for <30% of the studies, individual observations were weighted by the experimental replications^64^:

3 $${W}_{r}=({N}_{t}\times {N}_{c})/({N}_{t}+{N}_{c})$$

where *W*_*r*_ is the weight associated with each ln*RR* observation, and *N*_*t*_ and *N*_*c*_ are the number of replicates for the legume- and non-legume-based cropping system treatments, respectively. When yield values for treatment or control equaled zero and thereby indicated crop failure or experimental error, observations were excluded.

#### Variable importance

To identify the potentially relevant moderators in predicting the yield benefit of legume-based cropping system, a meta-regression model was fitted with 21 modifiers (Supplementary Table 3) as predictors using the R package *metaforest*^65^. The approach is adapted from the machine-learning algorithm “random forest” by incorporating the weight of each experimental observation to the bootstrap sampling. Thus, *metaforest* is robust to overfitting and considers non-linear relationships between moderators and the response variable, and their interactions^65,66^. We ranked the individual input factors according to their relative importance for predicting yield. Variable importance values were calculated based on the Gini impurity index that captures the variable importance for estimating the value of the target variable (here; the yield of the main crop) across all of the trees that make up the forest. If the variable is useful, it tends to split mixed labeled nodes into pure single class nodes^67^.

Exploratory moderator analysis was conducted based on the following steps. First, variable pre-selection was applied in *metaforest* using the *preselect* function. A recursive algorithm was used with 10,000 iterations and 100 replications. Variables with consistent negative importance were dropped using the *preselect_vars* function in metaforest. Variables with positive importance were retained for next-model optimization. Second, tuning parameters of *metaforest* were optimized using the *train* function from the *caret* package^68^. The optimal model was selected based on 10-fold cross-validation with minimized root-mean-square error (RMSE).

The metaforest models were fitted with different numbers of moderators by removing the variables containing missing values. This led to the selection of the following parameters for the total dataset with 18 moderators: uniform weighting metaforest, four candidate moderators available at each split, six as minimum node size. The estimated explained variance in out-of-bootstrap (${R}_{{oob}}^{2}$) cases and during cross-validation (${R}_{{cv}}^{2}$) ranged from 0.462 to 0.505 and 0.459 to 0.505, respectively, indicating that the optimized metaforest model had good predictive performance (Supplementary Table 5). The relative importance of included moderators in each dataset is shown in Fig. 2b and Supplementary Fig. 1. These results indicated that the importance of subsequent crop residue management, pre-crop residue management, and irrigation were relatively low in determining the legume pre-crop effect on yield. Therefore, the total dataset (11,768 paired observations) with 18 predictors was used in the following meta-analysis.

Through the above analyses, we found that the legume pre-crop effect was predominantly explained by the N fertilizer rate and crop diversity over a broad range of soil, climate, and management factors. Legume pre-crop effect on main crop yield was thus modeled as follows:

4 $${{{{\mathrm{ln}}}}}\,RR={\beta }_{0}+{\beta }_{1}\cdot N+{\beta }_{2}\cdot D+{\beta }_{3}\cdot N\times D+{\pi }_{{{{{\rm{study}}}}}}+\varepsilon$$

where *N* and *D* are N fertilizer rate applied on main crops and crop diversity, respectively. *β*_*i*_, *π*_study_, and *ε* are the mean effects, the random estimation error associated with the study, and sampling error, respectively. We conducted the analysis using the restricted maximum likelihood estimation with the *lme4* package^69^. Because most studies contributed more than one response ratio, the potential non-independency of studies was accounted for by including ‘study’ as a random factor. Our analysis indicated that many of our models violated the assumption of normality based on the Shapiro–Wilk test on model residuals. We therefore bootstrapped the fitted coefficients by 1000 iterations^64^. Legume pre-crops and the following main crops formed 170 cropping sequences; for ease of interpretation, only the first 60 major cropping sequences with number of observations >20 are shown (Fig. 5). We analyzed the potential for publication bias to influence our results using Fail-Safe N Analysis^70^. The fail-safe number (Rosenberg’s Nfs) for the database is 2,304,804, which is >39-fold above the threshold of 58,840 (5 × *n* + 10), which is considered the threshold for robust mean effect size. The funnel plot was sufficiently symmetrical (Supplementary Fig. 8). Thus, we did not find significant publication bias that might bias our results. The yield effects of legume pre-crops were considered significant if the 95% confidence interval did not overlap with zero. The mean effect sizes between groups were significantly different if their 95% CIs did not overlap with each other. To ease interpretation, the ln*RR* was finally back-transformed to generate the percentage change of rotation effect on yield, with percentage change = [exp (ln*RR*) –1] × 100. All statistical analyses were performed in R4.0.3^71^.

### Reporting summary

Further information on research design is available in the Nature Research Reporting Summary linked to this article.

## Supplementary information

Supplementary Information

Reporting Summary

Description of Additional Supplementary Files

Supplementary Data 1

## Data Availability

The source data underlying Figs. 1–5 and Supplementary Figs. 1–8 and Supplementary Tables 1–5 have been deposited in Figshare (10.6084/m9.figshare.20290923). The missing climate variables were extracted from the WorldClim database (https://worldclim.org/data/worldclim21.html). The missing soil physicochemical properties were extracted from the HWSD database (http://www.fao.org/soils-portal/data-hub/soil-maps-and-databases/harmonized-world-soil-database-v12/en/).

## Code availability

The R scripts needed to reproduce the analysis have been deposited in Figshare (10.6084/m9.figshare.20290959).
