# Supplementary material for: Global systematic review with meta-analysis reveals yield advantage of legume-based rotations and its drivers
Source: Nat Commun. 2022 Aug 22;13:4926. doi: 10.1038/s41467-022-32464-0 (PMC9395539; doi:10.1038/s41467-022-32464-0)
Supplement: Supplementary file 1 — Supplementary Information [file 41467_2022_32464_MOESM1_ESM.pdf]

Supplementary Information for

Global systematic review with meta-analysis reveals yield advantage of legume-based rotations and its drivers

Jie Zhao[1], Ji Chen[2], Damien Beillouin[3], Hans Lambers[4], Yadong Yang[1], Pete Smith[5],

Zhaohai Zeng[1, *], Jørgen E. Olesen[2], Huadong Zang[1, *]

[1] *College of Agronomy and Biotechnology, China Agricultural University, Beijing, China*

[2] *Department of Agroecology, Aarhus University, Blichers Allé 20, 8830 Tjele, Denmark*

[3] *CIRAD, UPR HORTSYS, Montpellier, France*

[4] *School of Biological Sciences and Institute of Agriculture, The University of Western Australia, 35 Stirling Highway, Crawley (Perth), WA 6009, Australia*

[5] *Institute of Biological and Environmental Sciences, University of Aberdeen, 23 St Machar Drive, AB24 3UU Aberdeen, UK*

*Corresponding author

Zhaohai Zeng (zengzhaohai@cau.edu.cn)

Huadong Zang (zanghuadong@cau.edu.cn)

Supplementary Figures

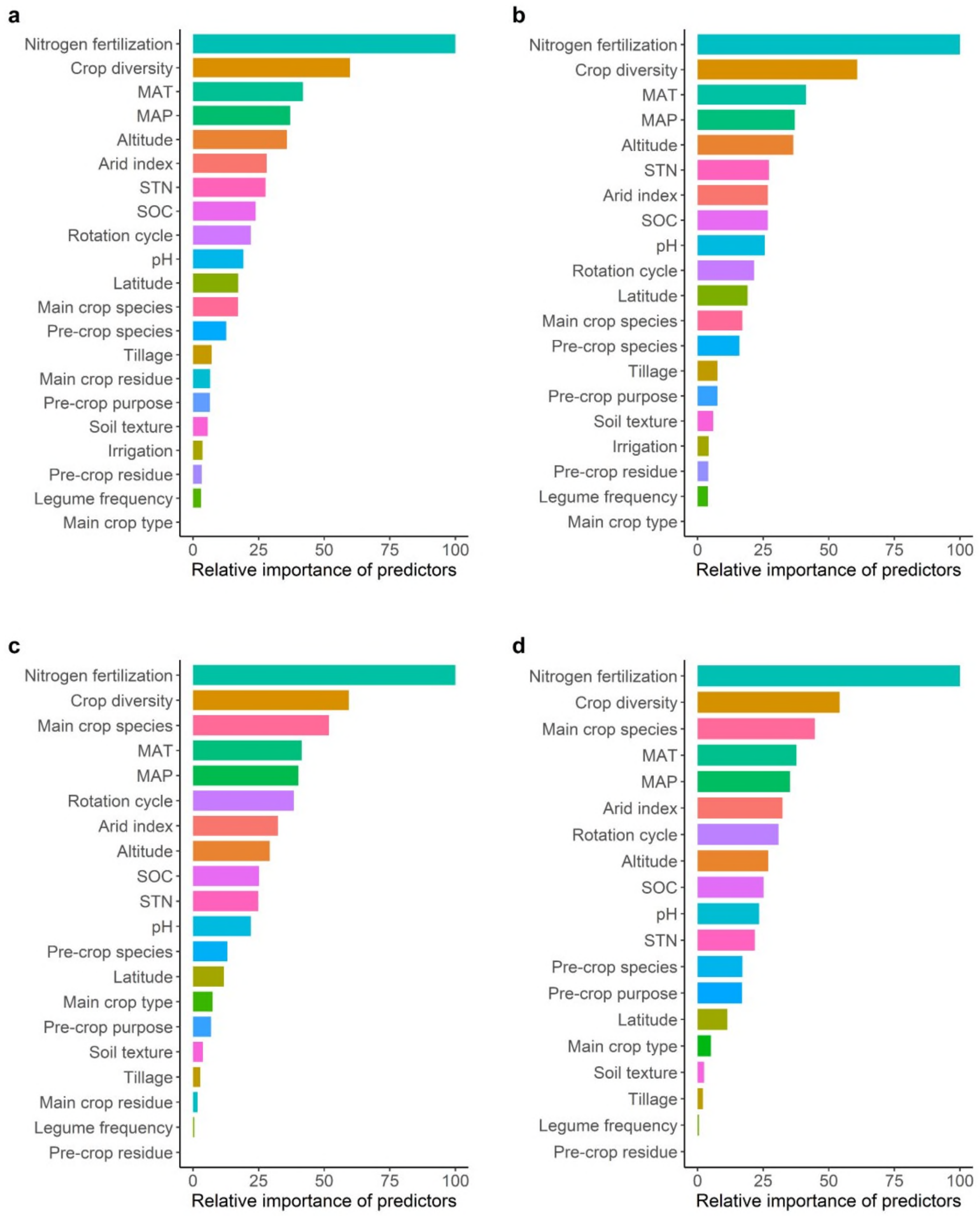

Supplementary Figure 1. Relative importance of 21 predictors determining the effect of legume pre-crops on main crop yield. The meta-forest models were fitted with different numbers of moderators by removing the variables containing missing values. **a**, 21 predictors, *n* = 5676 (208 studies). **b**, 20 predictors (no variable 'main crop residue management'), *n* = 6435 (252 studies). **c**, 20 predictors (no variable 'irrigation'), *n* = 8256 (297 studies). **d**, 19 predictors (no variable 'main crop residue management' and 'irrigation'), *n* = 9751 (378 studies). The meta-forest analysis demonstrated that predictors 'main crop residue management' and 'irrigation' had a relatively small impact, whereas N fertilizer rate and crop diversity were always the most important predictors describing the response of main crop yield to legume-based crop rotation.

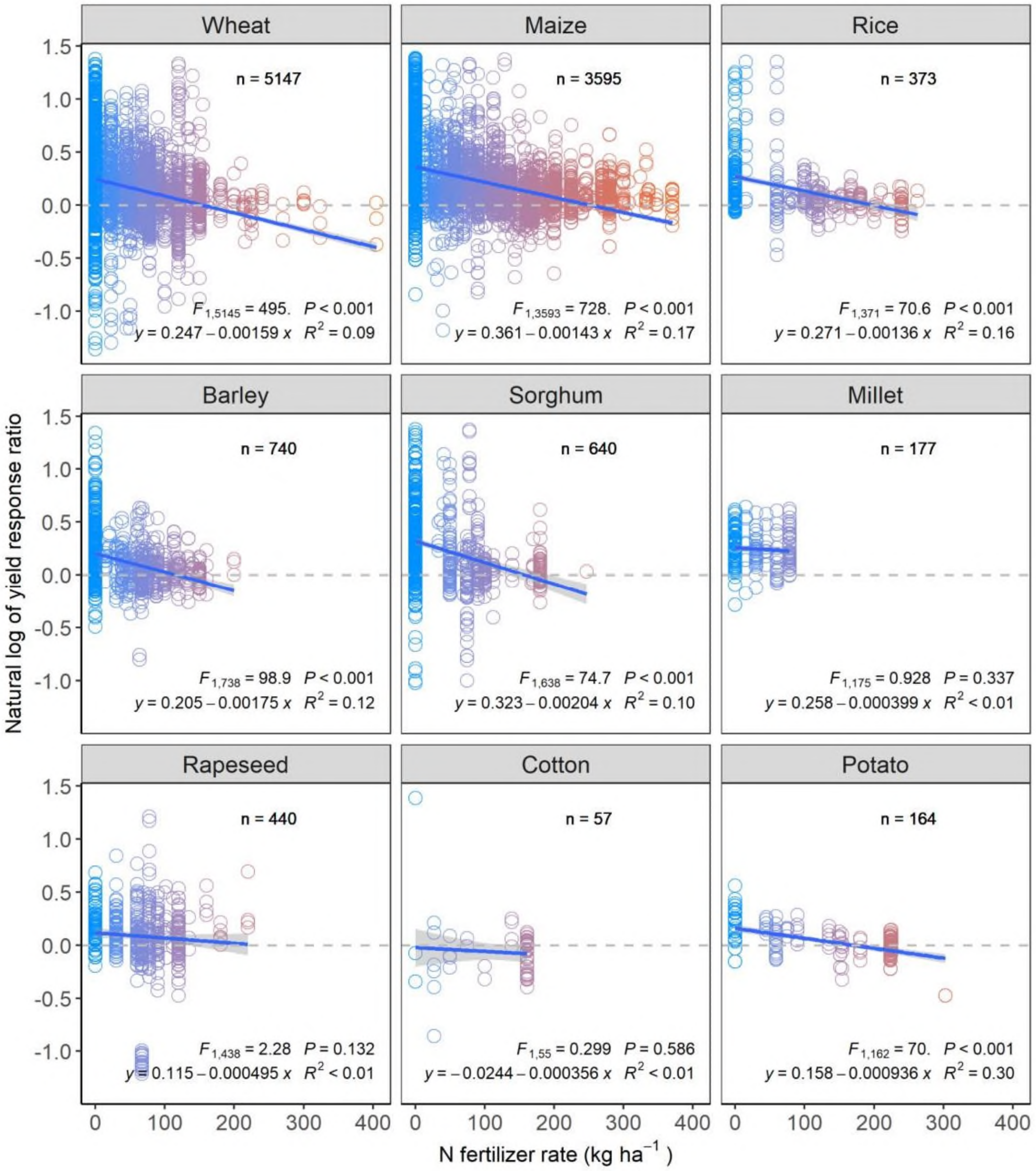

Supplementary Figure 2. Natural log of main crop yield ratios (main crop cultivated after legume pre-crops/main crop cultivated after non-legume pre-crops) as a function of the nitrogen (N) fertilizer rate (kg N ha^{-1}) applied to main crops. Linear relationships of N fertilizer rate and the natural log yield ratios of specific main crop species were fitted using a linear regression model. The blue curve in each panel indicates the average responses with their 95% confidence intervals indicated by shading.

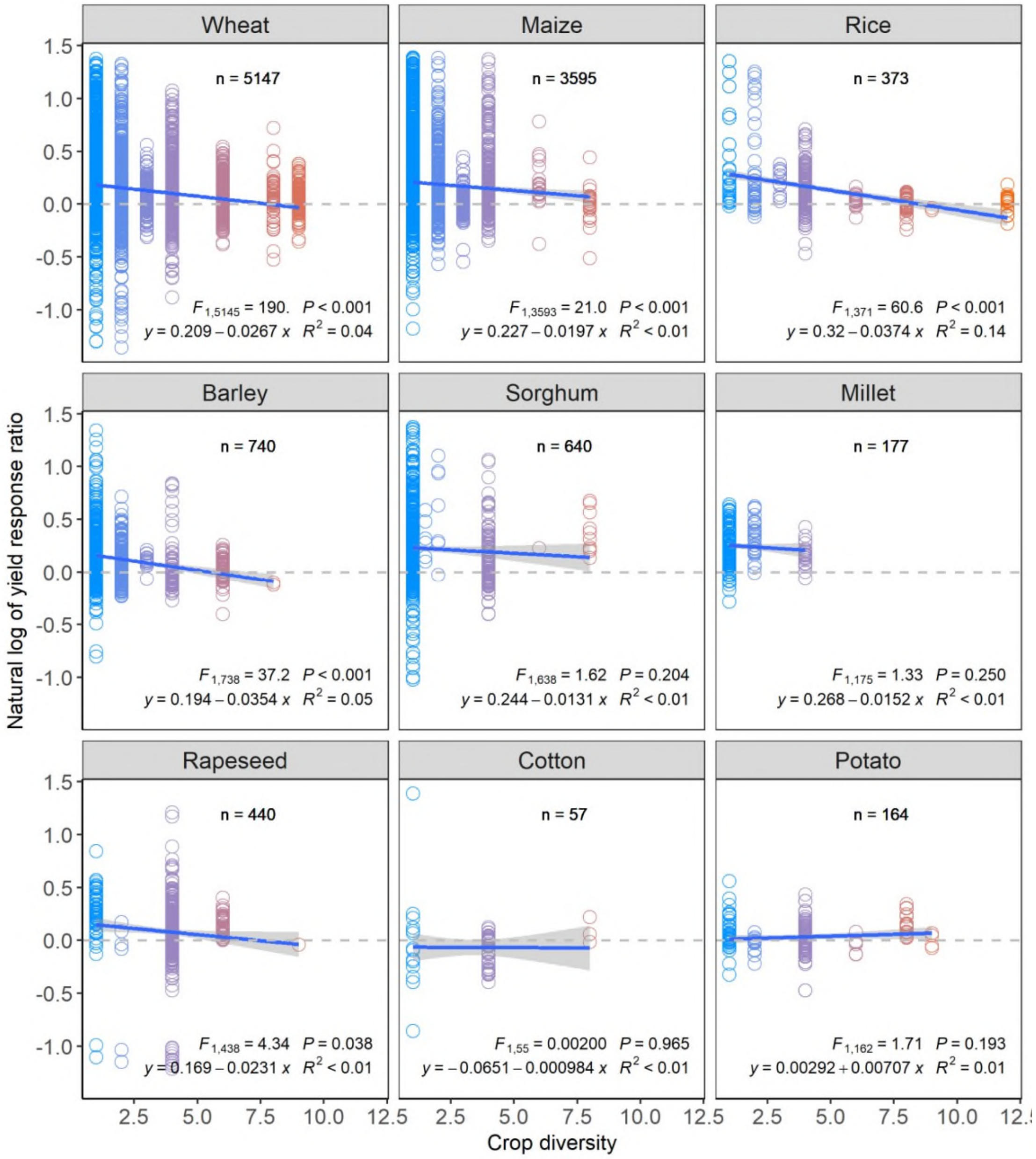

Supplementary Figure 3. Natural log of main crop yield ratios (main crop cultivated after legume pre-crops/main crop cultivated after non-legume pre-crops) as a function of the crop diversity (defined as number of crop species × number of crop functional groups × number of crop species per year) of main cropping systems. Linear relationships of crop diversity and the natural log yield ratios of specific main crop species were fitted using a linear regression model. The blue curve in each panel indicates the average responses with their 95% confidence intervals indicated by shading.

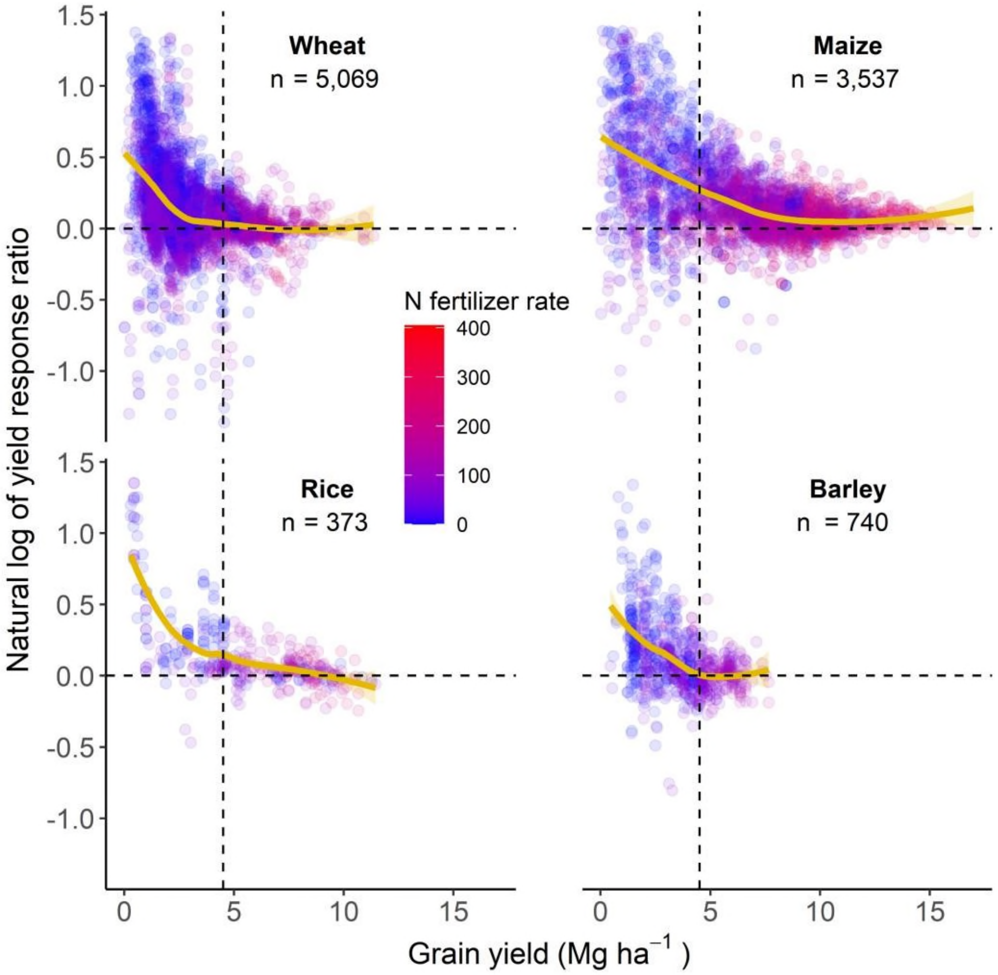

Supplementary Figure 4. The log yield response ratio (yield of crops following legumes vs. nonlegumes) decreased with increasing yield levels with a more pronounced pre-crop effect at low N fertilizer rate. The dashed horizontal and vertical lines were drawn at ln*RR* = 0 and averaged grain yield of each crop, respectively. The solid lines and shading area represent the smoothed conditional average response and their 95% confidence intervals fitted by local weighted regression using “loess” function in R.

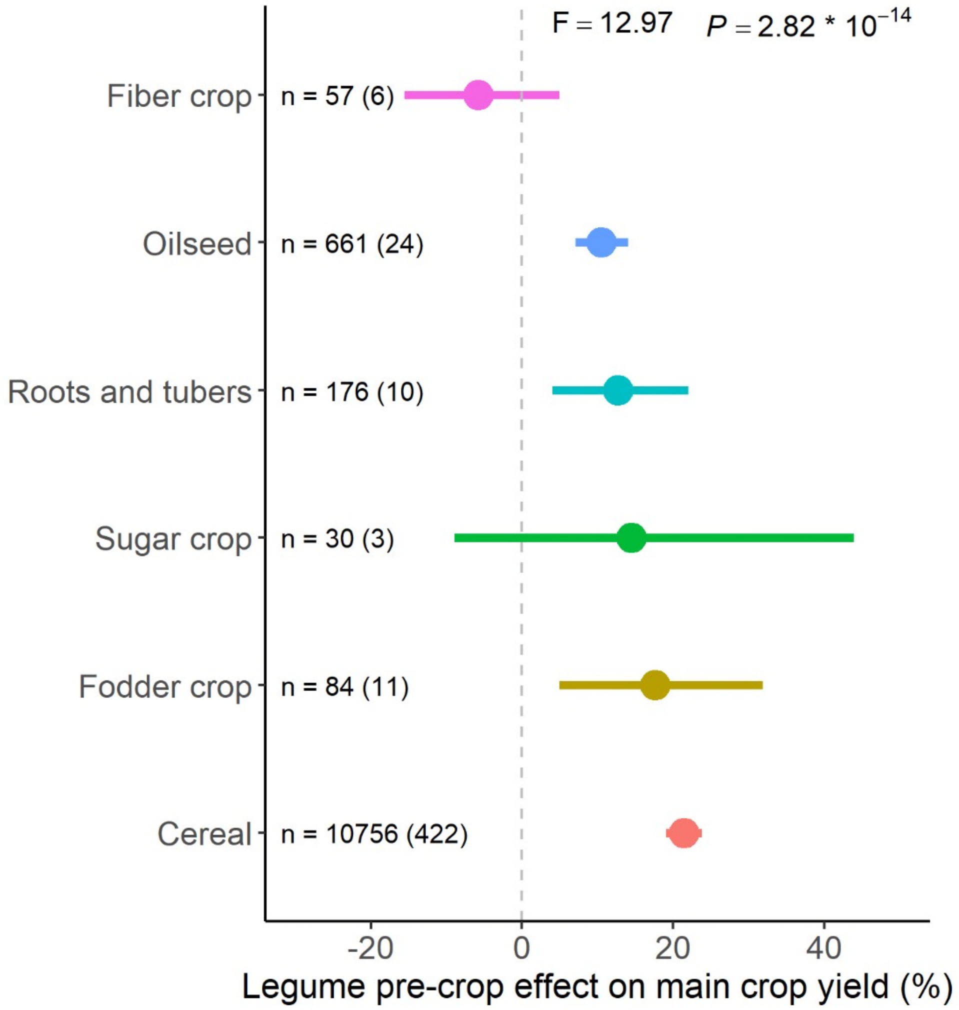

Supplementary Figure 5. Comparison of main crop yield in legume- versus non-legume-based cropping systems among functional groups of main crop species. Dots and horizontal bars represent overall effect sizes from a meta-analysis and 95% confidence intervals around the means. For each functional group, the number of observations is shown, with the number of studies in parentheses. Linear mixed-effect models used Satterthwaite approximation for degrees of freedom. *P* values, derived from the linear mixed model with the functional group as the fixed effect and study as the random effect, represent the significance of the difference in yield advantages among main crop types.

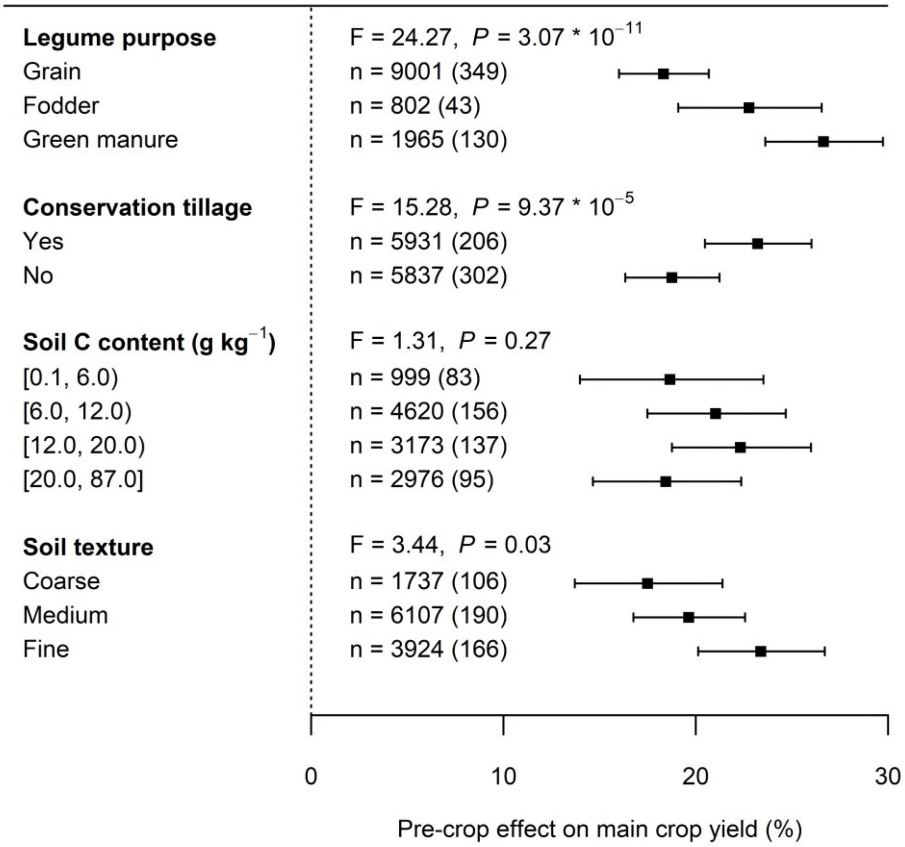

Supplementary Figure 6. Meta-analysis of legume pre-crop effects on main crop yield mediated by management and soil factors. For each sub-group, the number of observations is shown, with the number of studies in parentheses. Dots and horizontal bars represent overall effect sizes from a meta-analysis and 95% confidence intervals (CIs). Linear mixed-effect models used Satterthwaite approximation for degrees of freedom. Error bar does not overlap 0 means significant increase at $P < 0.05$. The mean effect sizes between groups were significantly different if their 95% CIs did not overlap with each other. Effect sizes were calculated and analyzed using log response-ratios, which were back-transformed and converted to percentage change.

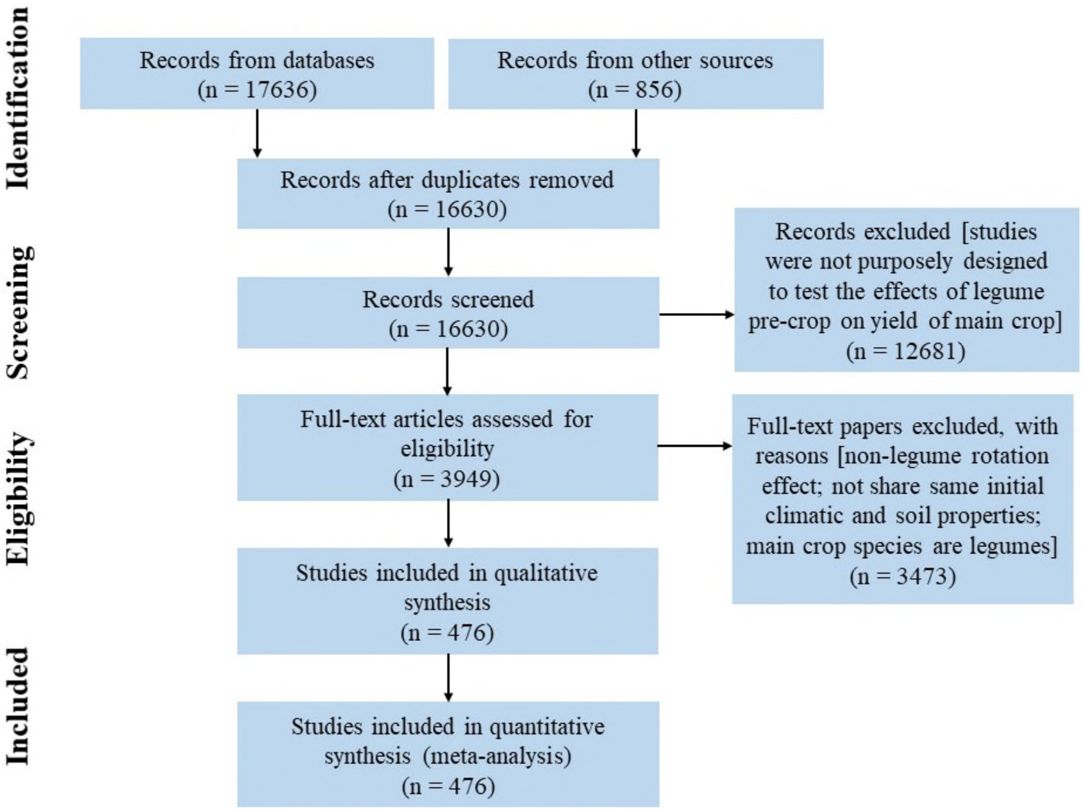

Supplementary Figure 7. PRISMA diagram showing the process of locating publications included in the present meta-analysis.

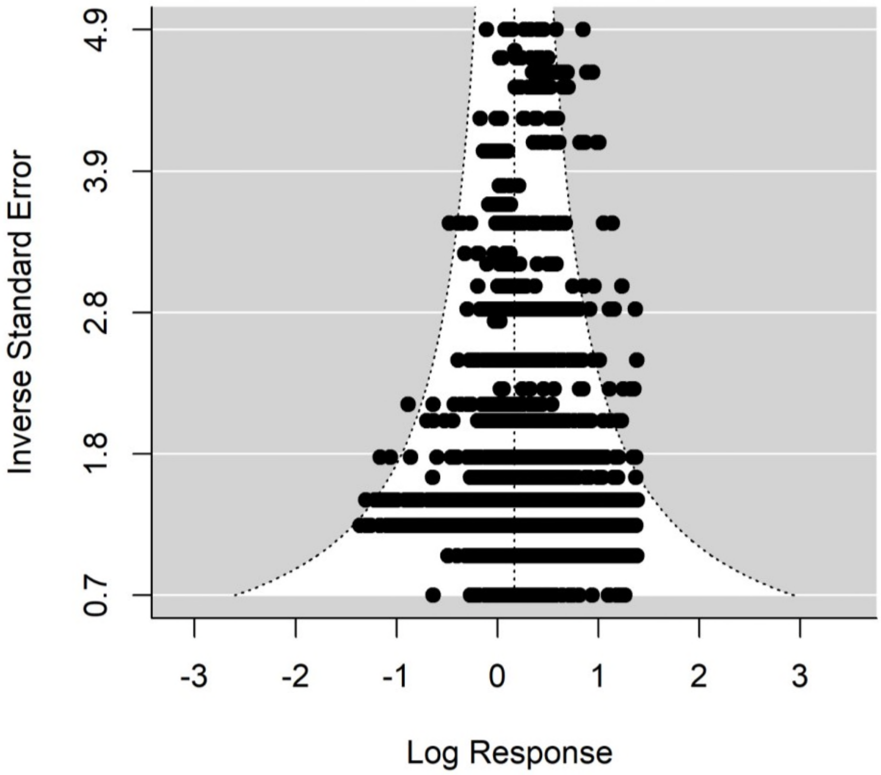

Supplementary Figure 8. Funnel plots for log-response ratios of yield under legume- versus nonlegume-based cropping system.

Supplementary Table

Supplementary Table 1. Legume crop species, sample size, and their pre-crop effect on main crop yield, irrespective of main crop species

Common name	Scientific name	Number of data pairs	Number of studies	Effect on yield (%)	CI %
Soybean	*Glycine max*	3040	175	15.9	13.1 to 18.6
Pea	*Pisum sativum*	1866	69	21.4	18.1 to 24.6
Alfalfa	*Medicago sativa*	972	48	18.8	15.2 to 22.3
Faba bean	*Vicia faba*	936	31	21.5	17.6 to 25.4
Chickpea	*Cicer arietinum*	793	30	12.8	9.2 to 16.3
Clover	*Trifolium*	708	40	23.5	19.6 to 27.4
Lentil	*Lens culinaris*	671	29	15.4	11.8 to 19.0
Mixture	-	528	15	20.7	16.1 to 25.3
Vetch	*Vicia*	387	50	28.5	23.9 to 33.0
Lupine	*Lupinus*	381	22	29.3	24.2 to 34.4
Common bean	*Phaseolus vulgaris*	326	17	14.5	9.5 to 19.5
Cowpea	*Vigna unguiculata*	305	32	28.7	22.8 to 34.5
Mungbean	*Vigna radiata*	194	21	14.2	8.7 to 19.7
Peanut	*Arachis hypogaea*	132	21	19.2	12.0 to 26.3
Pigeon pea	*Cajanus cajan*	126	15	32.4	23.8 to 41.0
Lablab	*Lablab purpureus*	98	8	37.2	28.1 to 46.2
Sesbania	*Sesbania*	59	8	30.3	19.5 to 41.1
Black gram	*Vigna mungo*	37	6	16.0	3.7 to 28.3
Mucuna	*Mucuna*	37	7	58.6	43.1 to 74.1
Sunn hemp	*Crotalaria juncea*	37	5	25.7	12.6 to 38.8
Medic	-	31	6	19.0	11.3 to 26.7
Crotalaria	*Crotalaria*	24	4	60.4	33.6 to 87.1
Acacia angustissima	*Acaciella angustissima*	12	2	26.3	2.4 to 50.1
Desmanthus virgatus	*Desmanthus virgatus*	11	1	-2.3	-20.3 to 15.8
Misc. forage	-	10	1	67.7	20.0 to 115.3
Lima bean	*Phaseolus lunatus*	9	1	33.9	2.0 to 65.8
Jackbean	*Canavalia ensiformis*	7	1	68.1	23.6 to 112.6
Winged bean	*Psophocarpus tetragonolobus*	6	1	53.5	11.5 to 95.4
Cluster bean	*Cyamopsis tetragonoloba*	4	2	1.5	-18.5 to 21.5
Lathyrus	*Lathyrus*	4	2	30.2	11.4 to 49.0

Centrosema pascuorum	*Centrosema pascuorum*	3	1	56.7	-12.8 to 126.1
Chamaecrista rotundifolia	*Chamaecrista rotundifolia*	3	1	145.8	36.9 to 254.7
Stylosanthes	*Stylosanthes*	3	1	85.9	3.5 to 168.2

Supplementary Table 2. Main crop species, sample size, and their yield response to legume precrops, irrespective of legume crop species.

Common name	Scientific name	Number of data pairs	Number of studies	Effect on yield (%)	CI %
Wheat	*Triticum aestivum*	5147	137	14.6	11.4 to 17.7
Maize	*Zea mays*	3595	175	28.9	25.5 to 32.2
Barley	*Hordeum vulgare*	740	36	14.9	10.4 to 19.4
Sorghum	*Sorghum bicolor*	640	33	28.4	22.8 to 34.0
Rapeseed	*Brassica napus*	440	11	3.9	0.0 to 7.8
Rice	*Oryza sativa*	373	40	18.8	11.9 to 25.6
Millet	*Panicum miliaceum*	177	10	17.0	10.1 to 23.9
Potato	*Solanum tuberosum*	164	9	13.6	4.0 to 23.1
Sunflower	*Helianthus*	82	3	21.8	14.0 to 29.5
Cotton	*Gossypium*	57	6	-1.3	-12.0 to 9.5
Oat	*Avena sativa*	49	4	-0.2	-8.5 to 8.2
Buckwheat	*Fagopyrum esculentum*	42	1	5.4	-3.5 to 14.2
Safflower	*Carthamus tinctorius*	42	1	3.4	-5.6 to 12.4
Crambe	*Crambe*	41	1	-1.2	-9.9 to 7.6
Flax	*Linum usitatissimum*	41	1	1.9	-7.1 to 10.8
Triticale	*Triticosecale*	40	3	26.5	15.3 to 37.7
Sugarcane	*Saccharum*	26	3	8.9	-20.3 to 38.1
Rye	*Secale cereale*	20	5	26.5	9.2 to 43.8
Cuphea	*Cuphea*	12	1	18.9	2.5 to 35.2
Cassava	*Manihot esculenta*	9	1	-9.7	-44.8 to 25.4
Sudan grass	*Sorghum sudanense*	8	1	30.6	-17.4 to 78.5
Quinoa	*Chenopodium quinoa*	4	2	-7.5	-32.0 to 17.1
Sugar beet	*Beta vulgaris*	4	1	52.1	-7.6 to 111.8
Tobacco	*Nicotiana tabacum*	4	1	13.3	-35.3 to 61.8

Mustard	*Brassica juncea*	3	1	20.0	-6.2 to 46.1
Poa pratensis	*Poa pratensis*	3	1	21.2	-33.4 to 75.8
Sweet potato	*Ipomoea batatas*	3	1	4.5	-35.1 to 44.1
Teff	*Eragrostis tef*	2	1	16.4	-23.1 to 55.8

Supplementary Table 3. List of predictors used to examine the effect of legume-based rotations

Predictor	Class	Definition	Source
Latitude	Temperate<br>Subtropical<br>Tropical	30°-66°<br>20°-30°<br>0°-20°	Reported in papers, Google map
Altitude	-	Altitude of study site	Reported in papers
Mean annual temperature (MAT)	-	Long-term mean annual temperature	Reported in papers, WorldCli v 2.1
Mean annual precipitation (MAP)	-	Long-term mean annual precipitation	Reported in papers, WorldCli v 2.1
Arid index	-	MAP divided by potential evapotranspiration	Reported in papers, WorldCli v 2.1
pH	-	Soil pH of top soil	Reported in papers, HWSD v 1.2
Soil organic carbon (SOC)	-	Soil organic carbon content in top soil (g kg^{-1})	Reported in papers, HWSD v 1.2
Total nitrogen (TN)	-	Soil total nitrogen concentration in topsoil (g kg^{-1})	Reported in papers, HWSD v 1.2
Soil texture	Coarse<br>Medium<br>Fine	Following USDA textural classification of soils	Reported in papers, HWSD v 1.2
Pre-crop species	-	Preceding legume crop species	Reported in papers
Pre-crop purpose	Seed<br>Green manure<br>Fodder	Preceding legume crop purpose	Reported in papers
Pre-crop residue management	Retain<br>Remove		Reported in papers
Main crop species	-	Subsequent main crop species	Reported in papers
Main crop type	Cereal Roots and tubers<br>Oilseed<br>Sugar crop<br>Fiber crop<br>Fodder crop	Following FAO definitions	

Main cropping system diversity	-	Crop diversity of the main cropping system calculated using equation (1)	
Nitrogen fertilization	-	Nitrogen fertilization rate on main crop (kg ha^{-1})	Reported in papers
Irrigation	Irrigated<br>Rainfed	Irrigation or not for main crop	Reported in papers
Conservation tillage	Yes<br>No	Conservation tillage or not for main crop	Reported in papers
Main crop residue management	Retain<br>Remove	Subsequent main crop residue management	Reported in papers

WorldCli v 2.1: WorldClim version 2.1 climate data for 1970-2000 (https://worldclim.org/data/worldclim21.html); HWSD v 1.2: Harmonized World Soil Database v 1.2 (http://www.fao.org/soils-portal/data-hub/soil-maps-anddatabases/harmonized-world-soil-database-v12/en/)

Supplementary Table 4. Description of paired cropping sequences in the initial cropping system and after legume inclusion and the example for the calculation of crop diversity of the initial cropping system.

**Initial cropping system (nonlegume)**	**After legume inclusion (legume-based)**	**Number of crop species**	**Number of crop functional groups**	**Number of crops per year**	**Crop diversity**	**References**
**wheat**-wheat	**pea**-wheat	1	1	1	1	Bonciarelli et al., 2016
**maize**-wheat	**pea**-wheat	2	1	1	2	Bonciarelli et al., 2016
**maize**-maize/wheat	**soybean**maize/wheat	2	1	1.5	3	Gao et al., 2014
**sunflower**-wheat	**pea**-wheat	2	2	1	4	Bonciarelli et al., 2016
**timothy**-rye-wheatbarley	**clover**-rye-wheatbarley	4	1	1	4	Ingver et al., 2018
**sunflower**-maizewheat	**soybean**-maizewheat	3	2	1	6	Studdert & Echeverria, 2000
**rapeseed**-barleywheat	**vetch**-barleywheat	3	2	1	6	Álvaro-Fuentes et al., 2009
**radish**/wheat	**clover**/wheat	2	2	2	8	Cicek et al., 2014
**oat**-barleyrapeseed-wheat	**pea**-wheatrapeseed-wheat	4	2	1	8	Christen et al., 1992

**potato**-wheat/maize	**peanut**wheat/maize	3	2	1.5	9	Wang et al., 2020
**buckwheat**sunflower-wheat	**chickpea**sunflower-wheat	3	3	1	9	Tanaka et al., 2010
**rapeseed**/rice/rice	**milkvetch**/rice/rice	2	2	3	12	Qaswar et al., 2019
**rapeseed**-maizewheat-tobacco	**soybean**-maizewheat-tobacco	4	3	1	12	Butorac et al., 1999

'-', annual cropping; '/', multiple cropping; paired initial cropping system (non-legume cropping system) and after legume inclusion (legume-based cropping system) in a cropping sequence are shown in boldface.

Supplementary Table 5. Performance of the metaforest models fitted with different numbers of moderators by removing the variables containing missing values.

Number of variables	Removed variable	Number of data pairs	Number of studies	RMSE	R_{oob2}	R_{cv2}
21	/	5,676	208	0.1957	0.5053	0.5051
20	main crop residue management	6,435	252	0.1941	0.4987	0.4932
20	Irrigation	8,256	297	0.2024	0.5046	0.4996
19	Main crop residue management, irrigation	9,751	378	0.2033	0.5135	0.4992
18	Main crop residue management, irrigation, and pre-crop residue management	11,768	462	0.2127	0.4615	0.4592

Root mean square error (RMSE) was used to select the optimal model using the smallest value; $R_{oob}{}^2$, out of bag R^2 of the final model; $R_{cv}{}^2$, cross-validated R^2 of the final model.

Supplementary references

1 Alvaro-Fuentes, J., Lampurlanes, J. & Cantero-Martinez, C. Alternative crop rotations under Mediterranean no-tillage conditions: biomass, grain yield, and water-use efficiency. *Agron. J.* **101**, 1227-1233, doi:10.2134/agronj2009.0077 (2009).

2 Bonciarelli, U. *et al.* Long-term evaluation of productivity, stability and sustainability for cropping systems in Mediterranean rainfed conditions. *Eur. J. Agron.* **77**, 146-155, doi:10.1016/j.eja.2016.02.006 (2016).

3 Butorac, A. *et al.* Results of long-term experiments with growing flue-cured tobacco (Nicotiana tabacum L.) in monoculture and different types of crop rotations. *J. Agron. Crop Sci.* **183**, 271-285, doi:10.1046/j.1439-037x.1999.00349.x (1999).

4 Christen, O., Sieling, K. & Hanus, H. The effect of different preceding crops on the development, growth and yield of winter wheat. *Eur. J. Agron.* **1**, 21-28, doi:10.1016/s11610301(14)80058-0 (1992).

5 Cicek, H., Entz, M. H., Martens, J. R. T. & Bullock, P. R. Productivity and nitrogen benefits of late-season legume cover crops in organic wheat production. *Can. J. Plant Sci.* **94**, 771783, doi:10.4141/cjps2013-130 (2014).

6 Gao, B. *et al.* Nitrous oxide and methane emissions from optimized and alternative cereal cropping systems on the North China Plain: A two-year field study. *Sci. Total Environ.* **472**, 112-124, doi:10.1016/j.scitotenv.2013.11.003 (2014).

7 Ingver, A. *et al.* Leguminous pre-crops improved quality of organic winter and spring cereals. *Biol. Agric. Hortic.* **35**, 46-60, doi:10.1080/01448765.2018.1509728 (2019).

8 Qaswar, M. *et al.* Long-term green manure rotations improve soil biochemical properties, yield sustainability and nutrient balances in acidic paddy soil under a rice-based cropping system. *Agronomy-Basel* **9**, doi:10.3390/agronomy9120780 (2019).

9 Studdert, G. A. & Echeverria, H. E. Crop rotations and nitrogen fertilization to manage soil organic carbon dynamics. *Soil Sci. Soc. Am. J.* **64**, 1496-1503, doi:10.2136/sssaj2000.6441496x (2000).

10 Tanaka, D. L., Liebig, M. A., Krupinsky, J. M. & Merrill, S. D. Crop sequence influences on sustainable spring wheat production in the northern Great Plains. *Sustainability* **2**, 36953709, doi:10.3390/su2123695 (2010).

11 Wang, L. *et al.* Effects of seven diversified crop rotations on selected soil health indicators and wheat productivity. *Agronomy-Basel* **10**, doi:10.3390/agronomy10020235 (2020).

Supplementary note: Publications used for the meta-analysis

1 Acharya, U., Chatterjee, A. & Daigh, A.L.M. Effect of subsurface drainage, crop rotation, and tillage on crop yield in Fargo clay soil. *J. Soil Water Conserv.* **74**, 456-465 (2019).

2 Adamou, A., Bationo, A., Tabo, R. & Koala, S. in *Advances in Integrated Soil Fertility Management in Sub-Saharan Africa: Challenges and Opportunities.* (ed A. Bationo, Waswa, B., Kihara, J., Kimetu, J.) 589-598 (Springer, 2007).

3 Adams, A. M. *et al.* Long-term effects of integrated soil fertility management practices on soil chemical properties in the Sahel. *Geoderma* **366**, doi:10.1016/j.geoderma.2020.114207 (2020).

4 Adeux, G., Giuliano, S., Cordeau, S., Savoie, J. & Alletto, L. Low-input maize-based cropping systems implementing IWM match conventional maize monoculture productivity and weed control. *Agriculture-Basel* **7**, 74 (2017).

5 Adiku, S.G.K., Jones, J.W., Kumaga, F.K. & Tonyigah, A. Effects of crop rotation and fallow residue management on maize growth, yield and soil carbon in a savannah-forest transition zone of Ghana. *J. Agric. Sci.* **147**, 313-322 (2009).

6 Adjei-Nsiah, S. et al. Farmers' agronomic and social evaluation of productivity, yield and N_2-fixation in different cowpea varieties and their subsequent residual N effects on a succeeding maize crop. *Nutr. Cycl. Agroecosystems* **80**, 199-209 (2008).

7 Adjei-Nsiah, S., Kuyper, T.W., Leeuwis, C., Abekoe, M.K. & Giller, K.E. Evaluating sustainable and profitable cropping sequences with cassava and four legume crops: Effects on soil fertility and maize yields in the forest/savannah transitional agro-ecological zone of Ghana. *Field. Crop. Res.* **103**, 87-97 (2007).

8 Agomoh, I.V., Drury, C.F., Phillips, L.A., Reynolds, W.D. & Yang, X. Increasing crop diversity in wheat rotations increases yields but decreases soil health. *Soil Sci. Soc. Am. J.* **84**, 170-181 (2020).

9 Agustin, E.O. et al. Role of indigo in improving the productivity of rainfed lowland rice-based cropping systems. *Exp. Agric.* **35**, 201-210 (1999).

10 Ahmad, T., Hafeez, F.Y., Mahmood, T. & Malik, K.A. Residual effect of nitrogen fixed by mungbean (*vigna radiata*) and blackgram (*Vigna Mungo*) on subsequent rice and wheat crops. *Aust. J. Exp. Agric.* **41**, 245-248 (2001).

11 Ai, C. et al. Distinct responses of soil bacterial and fungal communities to changes in fertilization regime and crop rotation. *Geoderma* **319**, 156-166 (2018).

12 Aiken, R.M., O Brien, D.M., Olson, B.L. & Murray, L. Replacing fallow with continuous cropping reduces crop water productivity of semiarid wheat. *Agron. J.* **105**, 199-207 (2013).

13 Akande, M.O., Makinde, E.A. & Adetunji, M.T. Response of maize and cowpea grown sequentially to application of phosphate rock in the humid tropics. *Commun. Soil Sci. Plant Anal.* **42**, 1027-1037 (2011).

14 Álvaro-Fuentes, J., Lampurlanés, J. & Cantero-Martínez, C. Alternative crop rotations under Mediterranean no-tillage conditions: Biomass, grain yield, and water-use efficiency. *Agron. J.* **101**, 1227-1233 (2009).

15 Anders, M. M. *et al.* The effect of rotation, tillage, fertility, and variety on rice grain yield and nutrient uptake. AAES Research Series **529**, 250-258 (2004).

16 Andrade, J.F., Poggio, S.L., Ermácora, M. & Satorre, E.H. Land use intensification in the rolling pampa, Argentina: Diversifying crop sequences to increase yields and resource use.

Eur. J. Agron. **82**, 1-10 (2017).

Anyanzwa, H. et al. Effects of conservation tillage, crop residue and cropping systems on
17 changes in soil organic matter and maize–legume production: A case study in Teso District.
Nutr. Cycl. Agroecosystems **88**, 39-47 (2010).

Ardjasa, W.S.R.A., Ando, H. & Kimura, M. Yield and soil erosion among cassava-based
18 cropping patterns in South Sumatra. *Soil Sci. Plant Nutr.* **47**, 101-112 (2001).

Arihara, J.H.N.A. & Karasawa, T. Effect of previous crops on arbuscular mycorrhizal
19 formation and growth of succeeding maize. *Soil Sci. Plant Nutr.* **46**, 43-51 (2000).

Armstrong, R.D. et al. Effects of long-term rotation and tillage practice on grain yield and
20 protein of wheat and soil fertility on a Vertosol in a medium-rainfall temperate environment.
Crop Past. Sci. **70**, 1 (2019).

Armstrong, R.D. et al. Legume and opportunity cropping systems in Central Queensland. 2.
21
Effect of legumes on following crops. *Aust. J. Agric. Resour. Econ.* **50**, 925-936 (1999).

Army, T. J. & Hide, J. C. Effects of green manure crops on dryland wheat production in the
22
Great Plains Area of Montana. *Agron. J.* **51**, 196–198 (1959).

Ashworth, A.J., Allen, F.L., Saxton, A.M. & Tyler, D.D. Long-term corn yield impacted by
23 cropping rotations and bio-covers under no-tillage. *Agron. J.* **108**, 1495-1502 (2016).

Ashworth, A.J., Owens, P.R. & Allen, F.L. Long-term cropping systems management
24 influences soil strength and nutrient cycling. *Geoderma* **361**, 114062 (2020).

Austenson, H.M., Wenhardt, A. & White, W.J. Effect of summer fallowing and rotation on
25 yield of wheat, barley and flax. *Can. J. Plant Sci.* **50**, 659-666 (1970).

Azevedo, D.M.P.D., Landivar, J., Vieira, R.M. & Moseley, D. The effect of cover crop and 26
crop rotation on soil water storage and on sorghum yield. *Pesqui Agropecu Bras* **34**, 391-398 (1999).

Badaruddin, M. & Meyer, D. W. Green-manure legume effects on soil nitrogen, grain yield,
27 and nitrogen nutrition of wheat. *Crop Sci.* **30**, 819-825 (1990).

Badaruddin, M. & Meyer, D.W. Forage legume effects on soil nitrogen and grain yield, and
28 nitrogen nutrition of wheat. *Agron. J.* **81**, 419-424 (1989).

Badaruddin, M. & Meyer, D.W. Grain legume effects on soil nitrogen, grain yield, and
29 nitrogen nutrition of wheat. *Crop Sci.* **34**, 1304-1309 (1994).

Bado, V., Bationo, A., Lompo, F., Cescas, M. & Sedogo, M. in *Advances in Integrated Soil* 30
Fertility Management in sub-Saharan Africa: Challenges and Opportunities. (ed A.
Bationo, Waswa, B., Kihara, J., Kimetu, J.) 171-177 (Springer, 2007).

Bagayoko, M., Mason, S.C. & Sabata, R.J. Effects of previous cropping systems on soil
31 nitrogen and grain sorghum yield. *Agron. J.* **84**, 862-868 (1992).

Bakhshandeh, S., Corneo, P.E., Mariotte, P., Kertesz, M.A. & Dijkstra, F.A. Effect of crop
32 rotation on mycorrhizal colonization and wheat yield under different fertilizer treatments.
Agric. Ecosyst. Environ. **247**, 130-136 (2017).

Bakht, J., Shafi, M., Jan, M.T. & Shah, Z. Influence of crop residue management, cropping
33 system and N fertilizer on soil N and C dynamics and sustainable wheat (*Triticum
Aestivum* L.) production. *Soil Tillage Res.* **104**, 233-240 (2009).

Baldock, J.O., Higgs, R.L., Paulson, W.H., Jackobs, J.A. & Shrader, W.D. Legume and

mineral N effects on crop yields in several crop sequences in the upper Mississippi Valley. *Agron. J.* **73**, 885-890 (1981).

Balkcom, K. S. & Reeves, D. W. Sunn-hemp utilized as a legume cover crop for corn production. *Agron. J.* **97**, 26–31 (2005).

Ballesta, A. & Lloveras, J.I.D.R. Nitrogen replacement value of alfalfa to corn and wheat under irrigated Mediterranean conditions. *Span J. Agric. Res.* **8**, 159-169 (2010).

Bavougian, C.M., Sarno, E., Knezevic, S. & Shapiro, C.A. Cover crop species and termination method effects on organic maize and soybean. *Biol. Agric. Hortic* **35**, 1-20 (2019).

Beckie, H. J. & Brandt, S. A. Nitrogen contribution of field pea in annual cropping systems. 1. Nitrogen residual effect. *Can. J. Plant Sci.* **77**, 311–322 (1997).

Bell, L.W., Lawrence, J., Johnson, B. & Peoples, M.B. New ley legumes increase nitrogen fixation and availability and grain crop yields in subtropical cropping systems. *Crop Past. Sci.* **68**, 11 (2017).

Bergerou, J.A., Gentry, L.E., David, M.B. & Below, F.E. Role of N_2 fixation in the soybean N credit in maize production. *Plant Soil* **262**, 383-394 (2004).

Biederbeck, V. O., Bouman, O. T., Campbell, C. A., Bailey, L. D. & Winkleman, G. E. Nitrogen benefits from four green-manure legumes in dryland cropping systems. *Can. J. Plant Sci.* **76**, 307–315 (1996).

Bitew, Y., Alemayehu, G., Adego, E. & Assefa, A. Impact of precursor crop and nitrogen fertilizer on the productivity of finger millet based cropping system and soil fertility in Lake Tana basin of Ethiopia. *J. Plant Nutr.* **43**, 1824-1839 (2020).

Blevins, R. L., Herbek, J. H. & Frye, W. W. Legume cover crops as a nitrogen source for no-till corn and grain sorghum. *Agron. J.* **82**, 769–772 (1990).

Bolton, E.F., Dirks, V.A. & Aylesworth, J.W. Some effects of alfalfa, fertilizer and lime on corn yield in rotations on clay soil during a range of seasonal moisture conditions. *Can. J. Plant Sci.* **56**, 21-25 (1976).

Bolton, E.F., Dirks, V.A. & McDonnell, M.M. The effect of drainage, rotation and fertilizer on corn yield, plant height, leaf nutrient composition and physical properties of Brookston clay soil in Southwestern Ontario. *Can. J. Plant Sci.* **62**, 297-309 (1982).

Bonciarelli, U. et al. Long-term evaluation of productivity, stability and sustainability for cropping systems in Mediterranean rainfed conditions. *Eur. J. Agron.* **77**, 146-155 (2016).

Boomsma, C.R. et al. Maize grain yield responses to plant height variability resulting from crop rotation and tillage system in a long-term experiment. *Soil Tillage Res.* **106**, 227-240 (2011).

Borase, D. N. *et al.* Long-term impact of grain legumes and nutrient management practices on soil microbial activity and biochemical properties. *Arch. Agron. Soil Sci.* **67**, 2015-2032, doi:10.1080/03650340.2020.1819532 (2021).

Borrell, A.N. et al. Fungal diversity associated with pulses and its influence on the subsequent wheat crop in the Canadian prairies. *Plant Soil* **414**, 13-31 (2017).

Borrelli, K. et al. Transition cropping system impacts on organic wheat yield and quality. *Renew. Agr. Food Syst.* **30**, 461-472 (2015).

Boyer, C.N., Roberts, R.K., Larson, J.A., McClure, M.A. & Tyler, D.D. Risk effects on optimal nitrogen rates for corn rotations in Tennessee. *Agron. J.* **107**, 896-902 (2015).

Bremer, E., Janzen, H.H., Ellert, B.H. & McKenzie, R.H. Soil organic carbon after twelve

years of various crop rotations in an Aridic Boroll. *Soil Sci. Soc. Am. J.* **72**, 970-974 (2008).

Brye, K.R. et al. Methane emissions as affected by crop rotation and rice cultivar in the Lower Mississippi River Valley, USA. *Geoderma Reg.* **11**, 8-17 (2017).

Bullied, W.J., Entz, M.H., Smith, J.S. & Bamford, K.C. Grain yield and N benefits to sequential wheat and barley crops from single-year alfalfa, berseem and red clover, chickling vetch and lentil. *Can. J. Plant Sci.***82**, 53-65 (2002).

Bundy, L.G., Andraski, T.W. & Wolkowski, R.P. Nitrogen credits in soybean-corn crop sequences on three soils. *Agron. J* **85**, 1061-1067 (1993).

Bunemann, E. K., Smithson, P. C., Jama, B., Frossard, E. & Oberson, A. Maize productivity and nutrient dynamics in maize-fallow rotations in western Kenya. *Plant Soil* **264**, 195-208, doi:10.1023/B:PLSO.0000047749.43017.fd (2004).

Bünemann, E.K., Heenan, D.P., Marschner, P. & McNeill, A.M. Long-term effects of crop rotation, stubble management and tillage on soil phosphorus dynamics. *Soil Res.* **44**, 611 (2006).

Buraczyńska, D., Ceglarek, F., Gąsiorowska, B., Zaniewicz-Bajkowska, A. & Płaza, A. Cultivation of wheat following pea and triticale/pea mixtures increases yields and nitrogen content. *Acta Agric. Scand B Soil Plant Sci.* **61**, 622-632 (2011).

Buxton, D.R. et al. Performance of sweet and forage sorghum grown continuously, double-cropped with winter rye, or in rotation with soybean and maize. *Agron. J.* **91**, 93-101 (1999).

Cai, S. et al. Rice rotation system affects the spatial dynamics of the diazotrophic community in paddy soil of the Yangtze delta, China. *Eurasian Soil Sci+* **52**, 696-706 (2019).

Cai, S., Pittelkow, C.M., Zhao, X. & Wang, S. Winter legume-rice rotations can reduce nitrogen pollution and carbon footprint while maintaining net ecosystem economic benefits. *J. Clean. Prod.* **195**, 289-300 (2018).

Calzarano, F. et al. Durum wheat quality, yield and sanitary status under conservation agriculture. *Agriculture* **8**, 140 (2018).

Campbell, C.A., Selles, F., Lafond, G.P., Biederbeck, V.O. & Zentner, R.P. Tillage-fertilizer changes: Effect on some soil quality attributes under long-term crop rotations in a thin Black Chernozem. *Can. J. Plant Sci.* **81**, 157-165 (2001).

Campbell, C.A., Zentner, R.P., Bowren, K.E., Townley-Smith, L. & Schnitzer, M. Effect of crop rotations and fertilization on soil organic matter and some biochemical properties of a Thick Black Chernozem. *Can. J. Plant Sci.* **71**, 377-387 (1991).

Campbell, C.A., Zentner, R.P., Selles, F., Biederbeck, V.O. & Leyshon, A.J. Comparative effects of grain lentil–wheat and monoculture wheat on crop production, N economy and N fertility in a Brown Chernozem. *Can. J. Plant Sci.***72**, 1091-1107 (1992).

Campiglia, E., Paolini, R., Colla, G. & Mancinelli, R. The effects of cover cropping on yield and weed control of potato in a transitional system. *Field. Crop. Res.* **112**, 16-23 (2009).

Cates, A.M. & Ruark, M.D. Soil aggregate and particulate C and N under corn rotations: Responses to management and correlations with yield. *Plant Soil* **415**, 521-533 (2017).

Chapman, A.L. & Myers, R. Nitrogen contributed by grain legumes to rice grown in rotation on the Cununurra soils of the Ord Irrigation Area, Western Australia. *Aust. J. Exp. Agric.* **27**, 155-163 (1987).

Chen, S. *et al.* Effect of various crop rotations on rice yield and nitrogen use efficiency in paddy–upland systems in southeastern China. *Crop J.* **6**, 576–588 (2018).

70 Chen, S. *et al.* The influence of the type of crop residue on soil organic carbon fractions: An 11-year field study of rice-based cropping systems in southeast China. *Agric. Ecosyst. Environ.* **223**, 261–269 (2016).

71 Cheruiyot, E. K., Mumera, L. M., Nakhone, L. N. & Mwonga, S. M. Effect of legumemanaged fallow on weeds and soil nitrogen in following maize (*Zea mays* L.) and wheat (*Triticum aestivum* L.) crops in the Rift Valley highlands of Kenya. *Aust. J. Exp. Agric.* **43**, 597–604 (2003).

72 Cheruiyot, E. K., Mumera, L. M., Nakhone, L. N. & Mwonga, S. M. Rotational effects of grain legumes on maize performance in the Rift Valley Highlands of Kenya. *Afr. crop sci. j. (Online)* **9**, 667–676 (2001).

73 Chikowo, R., Mapfumo, P., Leffelaar, P.A. & Giller, K.E. Integrating legumes to improve N cycling on smallholder farms in sub-humid Zimbabwe: Resource quality, biophysical and environmental limitations. *Nutr. Cycl. Agroecosystems* **76**, 219-231 (2007).

74 Chikowo, R., Mapfumo, P., Nyamugafata, P. & Giller, K.E. Maize productivity and mineral N dynamics following different soil fertility management practices on a depleted sandy soil in Zimbabwe. *Agric. Ecosyst. Environ.* **102**, 119-131 (2004).

75 Chikowo, R., Mapfumo, P., Nyamugafata, P. & Giller, K.E. Woody legume fallow productivity, biological N-2-fixation and residual benefits to two successive maize crops in Zimbabwe. *Plant Soil* **262**, 303-315 (2004).

76 Cicek, H., Entz, M.H., Martens, J.R.T. & Bullock, P.R. Productivity and nitrogen benefits of late-season legume cover crops in organic wheat production. *Can. J. Plant Sci.***94**, 771-783 (2014).

77 Clay, S.A., Clay, S.A., Aguilar, I. & Aguilar, I. Weed seedbanks and corn growth following continuous corn or alfalfa. *Agron. J.* **90**, 813-818 (1998).

78 Clegg, M.D. Effect of soybean on yield and nitrogen response of subsequent sorghum crops in Eastern Nebraska. *Field. Crop. Res.* **5**, 233-239 (1982).

79 Congreves, K.A., Hooker, D.C., Hayes, A., Verhallen, E.A. & Van Eerd, L.L. Interaction of long-term nitrogen fertilizer application, crop rotation, and tillage system on soil carbon and nitrogen dynamics. *Plant Soil* **410**, 113-127 (2017).

80 Cox, H.W., Kelly, R.M. & Strong, W.M. Pulse crops in rotation with cereals can be a profitable alternative to nitrogen fertiliser in Central Queensland. *Crop Past. Sci.* **61**, 752 (2010).

81 Crookston, K. R., Kurle, J. E. & Lueschen, E. Relative ability of soybean, fallow, and triacontanol to alleviate yield reductions associated with growing corn continously. *Crop Sci.* **28**, 145–147 (1988).

82 Crookston, R. K. & Kurle, J. E. Corn residue effect on the yield of corn and soybean grown in rotation. *Agron. J.* **81**, 229–232 (1989).

83 Cutforth, H.W., Jefferson, P.G., Campbell, C.A. & Ljunggren, R.H. Yield, water use, and protein content of spring wheat grown after six years of alfalfa, crested wheatgrass, or spring wheat in semiarid Southwestern Saskatchewan. *Can. J. Plant Sci.* **90**, 489-497 (2010).

Daigh, A.L.M. et al. Subsurface drainage nitrate and total reactive phosphorus losses in
84 bioenergy-based prairies and corn systems. *J. Environ. Qual.* **44**, 1638-1646 (2015).
Daigh, A.L.M. et al. Yields and yield stability of no-till and chisel-plow fields in the
85
Midwestern us corn belt. *Field. Crop. Res.* **218**, 243-253 (2018).
Dalal, R.C. et al. Sustaining productivity of a Vertisol at Warra, Queensland, with fertilisers,
86 no-tillage, or legumes. 5. Wheat yields, nitrogen benefits and water-use efficiency of chickpea-wheat rotation. *Aust. J. Exp. Agric.* **38**, 489-501 (1998).
Dalal, R.C. et al. Sustaining productivity of a Vertosol at Warra, Queensland, with fertilisers,
87 no-tillage or legumes. 8. Effect of duration of lucerne ley on soil nitrogen and water, wheat yield and protein. *Aust. J. Exp. Agric.* **44**, 1013-1024 (2004).
Dapaah, H.K.C.R. & Vyn, T.J. Nitrogen fertilization and cover crop effects on soil structural
88 stability and corn performance. *Commun. Soil Sci. Plant Anal.* **29**, 2557-2569 (1998).
Das, A. et al. Tillage and cropping sequence effect on physico-chemical and biological
89 properties of soil in Eastern Himalayas, India. *Soil Tillage Res.* **180**, 182-193 (2018).
Davis, J.R. et al. Ecological relationships of verticillium wilt suppression of potato by green
90 manures. *Am. J. Potato Res.* **87**, 315-326 (2010).
Davis, R.A., Huggins, D.R., Cook, J.R. & Paulitz, T.C. Nitrogen and crop rotation effects on
91 fusarium crown rot in no-till spring wheat. *Can. J. Plant Pathol.* **31**, 456-467 (2009).
De, R., Yogeswara Rao, Y. & Ali, W. Grain and fodder legumes as preceding crops affecting
92 the yield and N economy of rice. *J. Agric. Sci.* **101**, 463–466 (1983).
Debaeke, P. & Hilaire, A. Production of rainfed and irrigated crops under different crop
93 rotations and input levels in Southwestern France. *Can. J. Plant Sci.***77**, 539-548 (1997).
Delroy, N.D. & Bowden, J.W. Effect of deep ripping, the previous crop, and applied nitrogen
94 on the growth and yield of a wheat crop. *Aust. J. Exp. Agric.* **26**, 469-479 (1986). DeMaria, I.C., Nnabude, P.C. & de Castro, O.M. Long-term tillage and crop rotation effects
95 on soil chemical properties of a Rhodic Ferralsol in Southern brazil. *Soil Tillage Res.* **51**, 7179 (1999).
96 Díaz-Ambrona, C.H. & Mínguez, M.I. Cereal–legume rotations in a Mediterranean environment: Biomass and yield production. *Field. Crop. Res.* **70**, 139-151 (2001). Dick, W. A., Van Doren, D. M., Triplett, G. B. & Henry, J. E. Influence of long-term tillage
97 and rotation combinations on crop yields and selected soil parameters. I. Results obtained for a Mollic Ochraqualf soil. 30 (1986).
Dick, W. A., Van Doren, D. M., Triplett, G. B. & Henry, J. E. Influence of long-term tillage
98 and rotation combinations on crop yields and selected soil parameters. II. Results obtained for a Typic Fragiudalf soil. 30 (1986).
Ding, W., Hume, D.J., Vyn, T.J. & Beauchamp, E.G. N credit of soybean to a following corn
99 crop in Central Ontario. *Can. J. Plant Sci.***78**, 29-33 (1998).
Doolette, A. et al. Phosphorus uptake benefit for wheat following legume break crops in semi-
100 arid Australian farming systems. *Nutr. Cycl. Agroecosystems* **113**, 247-266 (2019). Doughton, J.A. & Mackenzie, J. Comparative effects of black and green gram (mung beans)
101 and grain sorghum on soil mineral nitrogen and subsequent grain sorghum yields on the Eastern Darling Downs. *Aust. J. Exp. Agric.* **24**, 244-249 (1984).
Dowler, C. C., Hauser, E. W. & Johnson, A. W. Crop-herbicide sequences on a southeastern
102
Coastal Plain soil. *Weed Sci.* **22**, 500–505 (1974).

Drury, C.F. & Tan, C.S. Long-term (35 years) effects of fertilization, rotation and weather on corn yields. *Can. J. Plant Sci.***75**, 355-362 (1995).

Drury, C.F. et al. Impacts of 49-51 years of fertilization and crop rotation on growing season nitrous oxide emissions, nitrogen uptake and corn yields. *Can. J. Plant Sci.* **94**, 421-433 (2014).

Dyke G. V. & Slope, D. B. Effects of previous legume and oat crops on grain yield and take-all in spring barley. *J. Agric. Sci.* **91**, 443–451 (1978).

Ebelhar, S. A., Frye, W. W. & Blevins, R. L. Nitrogen from legume cover crops for no-tillage corn. *Agron. J.* **76**, 51–55 (1984).

Eckert, D.J. Ridge planting for row crops on a poorly drained soil. 1. Rotation and drainage effects. *Soil Tillage Res.* **18**, 181-188 (1990).

Edwards, J.H., Thurlow, D.L. & Eason, J.T. Influence of tillage and crop rotation on yields of corn, soybean, and wheat. *Agron. J.* **80**, 76-80 (1988).

Eghball, B. & Varvel, G.E. Fractal analysis of temporal yield variability of crop sequences: implications for site-specific management. *Agron. J.* **89**, 851-855 (1997).

Ellis, J.R., Mason, S.C. & Roder, W. Grain sorghum-soybean rotation and fertilization influence on vesicular-arbuscular mycorrhizal fungi. *Soil Sci. Soc. Am. J.* **56**, 789-794 (1992).

Engström, L. & Lindén, B. Importance of soil mineral N in early spring and subsequent net N mineralisation for winter wheat following winter oilseed rape and peas in a milder climate. *Acta. Agric. Scand B Soil Plant Sci.* **59**, 402-413 (2009).

Entz, M.H., Bullied, W.J., Forster, D.A., Gulden, R. & Vessey, J.K. Extraction of subsoil nitrogen by alfalfa, alfalfa–wheat, and perennial grass systems. *Agron. J.* **93**, 495-503 (2001).

Espinoza, S. et al. Contribution of legumes to wheat productivity in Mediterranean environments of Central Chile. *Field. Crop. Res.* **133**, 150-159 (2012).

Espinoza, S., Ovalle, C. & Del Pozo, A. The contribution of nitrogen fixed by annual legume pastures to the productivity of wheat in two contrasting Mediterranean environments in Central Chile. *Field. Crop. Res.* **249**, 107709 (2020).

Evans, D.R., Williams, T.A. & Mason, S.A. Residual N effect of grazed white clover (*trifolium repens*)/ryegrass (*Lolium perenne*) swards on subsequent yields of spring barley. *J. Agric. Sci.* **118**, 175-178 (1992).

Evans, J. et al. Towards a more productive and sustainable cropping system in the democratic people's republic of Korea. I. Rice production. *J. Sustain. Agric.* **33**, 528-551 (2009).

Fan, R. et al. Tillage and rotation effects on crop yield and profitability on a black soil in Northeast China. *Can. J. Plant Sci* **92**, 463-470 (2012).

Faris, M.A., Smith, D.L. & Coulman, B.E. Plow down effects of different forage legume species, cultivars, cutting strategies and seeding rates on the yields of subsequent crops. *Plant Soil* **95**, 419-430 (1986).

Fischler, M. & Wortmann, C. S. Green manures for maize-bean systems in eastern Uganda: Agronomic performance and farmers' perceptions. *Agrofor. Syst.* **47**, 123-138, doi:10.1023/a:1006234523163 (1999).

Flower, K.C., Ward, P.R., Cordingley, N., Micin, S.F. & Craig, N. Rainfall, rotations and residue level affect no-tillage wheat yield and gross margin in a Mediterranean-type environment. *Field. Crop. Res.* **208**, 1-10 (2017).

Fordoński, G., Pszczółkowska, A., Krzebietke, S., Olszewski, J. & Okorski, A. Yield and
121 mineral composition of seeds of leguminous plants and grain of spring wheat as well as their residual effect on the yield and chemical composition of winter oilseed rape seeds. *J. Elem.* **20**, 827–838 (2015).

Fouli, Y. et al. Double cropping effects on forage yield and the field water balance. *Agric.*
122
Water Manag. **115**, 104-117 (2012).

Fox, R. H. & Piekielek, W. P. Fertilizer N equivalence of alfalfa, birdsfoot trefoil, and red
123 clover for succeeding corn crops. *J. Prod. Agric.* **317**, 313–317 (1988).

Franco, J.G. et al. Spring wheat yields following perennial forages in a semiarid no-till
124 cropping system. *Agron. J.* **110**, 2408-2416 (2018).

Franzluebbers, A.J., Hons, F.M. & Saladino, V.A. Sorghum, wheat and soybean production as
125 affected by long-term tillage, crop sequence and N fertilization. *Plant Soil* **173**, 55-65 (1995). Friberg, H., Persson, P., Jensen, D.F. & Bergkvist, G. Preceding crop and tillage system affect
126 winter survival of wheat and the fungal communities on young wheat roots and in soil. *FEMS Microbiol. Lett.* **366**, fnz189 (2019).

Gakale, L.P. & Clegg, M.D. Nitrogen from soybean for dryland sorghum. *Agron. J.* **79**, 1057-
127
1061 (1987).

Gan, Y.T. et al. Diversifying crop rotations with pulses enhances system productivity. *Sci.*
128
Rep. **5**, 14625 (2015).

Gan, Y.T. et al. Influence of diverse cropping sequences on durum wheat yield and protein in
129 the semiarid Northern Great Plains. *Agron. J.* **95**, 245-252 (2003).

Gan, Y.T., Hamel, C., Kutcher, H.R. & Poppy, L. Lentil enhances agroecosystem productivity
130 with increased residual soil water and nitrogen. *Renew. Agr. Food Syst.* **32**, 319-330 (2017). Gao, Y., et al. Interactive effects of tillage practices and cropping systems on the interannual 131 variation of soil carbon, nitrogen content and corn yield in Mollisol. *Soil and Crops* **9**, 323334 (2020).

Gaudin, A.C.M. et al. Increasing crop diversity mitigates weather variations and improves
132 yield stability. *PLoS One* **10**, e0113261 (2015).

Gaudin, A.C.M., Janovicek, K., Deen, B. & Hooker, D.C. Wheat improves nitrogen use 133 efficiency of maize and soybean-based cropping systems. *Agric. Ecosyst. Environ.* **210**, 1-10 (2015).

Gentry, L.F., Ruffo, M.L. & Below, F.E. Identifying factors controlling the continuous corn
134 yield penalty. *Agron. J.* **105**, 295-303 (2013).

Gesch, R.W., Archer, D.W. & Forcella, F. Rotational effects of cuphea on corn, spring wheat,
135 and soybean. *Agron. J.* **102**, 145-153 (2010).

Ghosh, B.N., Khola, O.P.S., Bhattacharyya, R., Dadhwal, K.S. & Mishra, P.K. Effect of
136 potassium on soil conservation and productivity of maize/cowpea based crop rotations in the North-west Indian Himalayas. *J. Mt. Sci.* **13**, 754-762 (2016).

Ghosh, P. K. *et al.* Grain legume inclusion in cereal-cereal rotation increased base crop 137 productivity in the long run. *Exp. Agric.* **56**, 142-158, doi:10.1017/s0014479719000243 (2020).

138 Giambalvo *et al.* Nitrogen efficiency component analysis in wheat under rainfed Mediterranean conditions: effects of crop rotation and nitrogen fertilization. *Options Mediterraneennes. Serie A, Seminaires Mediterraneens* **60**, 169–173 (2004).

139 Gill, K.S. Crop rotations compared with continuous canola and wheat for crop production and fertilizer use over 6 yr. *Can. J. Plant Sci.* **98**, 1139-1149 (2018).

140 Giri, G. & De, R. Effect of preceding grain legumes on dryland pearl millet in NW India. *Exp. Agric.* **15**, 169-172 (1979).

141 Gong, M.Q. et al. Effects of nitrogen reduction on rice yield and soil nutrients under different rotation systems. *J. Anhui Agric. Sci.* **48**,152-156 (2020).

142 Goplen, J.J. et al. Economic performance of crop rotations in the presence of herbicide-resistant giant ragweed. *Agron. J.* **110**, 260-268 (2018).

143 Götze, P., Rücknagel, J., Wensch-Dorendorf, M., Märländer, B. & Christen, O. Crop rotation effects on yield, technological quality and yield stability of sugar beet after 45 trial years. *Eur. J. Agron.* **82**, 50-59 (2017).

144 Grace, P.R., Oades, J.M., Keith, H.A. & Hancock, T.W. Trends in wheat yields and soil organic carbon in the permanent rotation trial at the Waite Agricultural Research Institute, South Australia. *Aust. J. Exp. Agric.* **35**, 857-864 (1995).

145 Grageda-Cabrera, O.A. et al. Fertilizer dynamics in different tillage and crop rotation systems in a Vertisol in Central Mexico. *Nutr. Cycl. Agroecosystems* **89**, 125-134 (2011).

146 Griffin, T.S., Larkin, R.P. & Honeycutt, C.W. Delayed tillage and cover crop effects in potato systems. *Am. J. Potato Res.* **86**, 79-87 (2009).

147 Griffith, D.R., Kladivko, E.J., Mannering, J.V., West, T.D. & Parsons, S.D. Long-term tillage and rotation effects on corn growth and yield on high and low organic matter, poorly drained soils. *Agron. J.* **80**, 599-605 (1988).

148 Grover, K. K., Karsten, H. D. & Roth, G. W. Corn grain yields and yield stability in four long-term cropping systems. *Agron. J.* **101**, 940–946 (2009).

149 Guertal, E.A., Bauske, E.M. & Edwards, J.H. Crop rotation effects on sweet potato yield and quality. *J. Prod. Agric* **10**, 70-73 (1997).

150 Gul, I., Akinci, C., Doran, I., Kilic, H. & Baytekin, H. Effects of different previous crops and nitrogen rates on double cropping maize (*Zea mays* L.). *Asian J. Chem.* **20**, 2947 (2008).

151 Halvorson, A.D. & Reule, C.A. Irrigated corn and soybean response to nitrogen under no-till in Northern Colorado. *Agron. J* **98**, 1367-1374 (2006).

152 Hamblin, J., Delane, R., Bishop, A. & Adam, G. The yield of wheat following lupins: Effects of different lupin genotypes and management. *Aust. J. Agric. Res.* **44**, 645–649 (1993).

153 Haque, I. & Lupwayi, N.Z. Nitrogen fixation by annual forage legumes and its contribution to succeeding wheat in the Ethiopian Highlands. *J. Plant Nutr.* **23**, 963-977 (2000).

154 Hargrove, W.L. Winter legumes as a nitrogen source for no-till grain sorghum. *Agron. J.* **78**, 70-74 (1986).

155 Hargrove, W.L., Touchton, J.T. & Johnson, J.W. Previous crop influence on fertilizer nitrogen requirements for double-cropped wheat. *Agron. J.* **75**, 855-859 (1983).

156 Heenan, D.P. Effects of broad-leaf crops and their sowing time on subsequent wheat production. *Field. Crop. Res.* **43**, 19-29 (1995).

Heenan, D.P., Taylor, A.C., Cullis, B.R. & Lill, W.J. Long term effects of rotation, tillage and

157 stubble management on wheat production in Southern NSW. *Aust. J. Agric. Resour. Econ.* **45**, 93-117 (1994).

158 Helmers, M.J., Zhou, X., Baker, J.L., Melvin, S.W. & Lemke, D.W. Nitrogen loss on tile-drained Mollisols as affected by nitrogen application rate under continuous corn and cornsoybean rotation systems. *Can. J. Plant Sci* **92**, 493-499 (2012).

159 Hergert, G.W. et al. Cropping systems for stretching limited irrigation supplies. *J. Prod. Agric* **6**, 520-529 (1993).

160 Hesterman, O.B., Sheaffer, C.C., Barnes, D.K., Lueschen, W.E. & Ford, J.H. Alfalfa dry matter and nitrogen production, and fertilizer nitrogen response in legume-corn rotations. *Agron. J.* **78**, 19-23 (1986).

161 Hirzel, J. et al. Different residues affect wheat nutritional composition. *J. Soil Sci. Plant Nutr.* **20**, 75-82 (2020).

162 Holderbaum, J.F., Decker, A.M., Messinger, J.J., Mulford, F.R. & Vough, L.R. Fall-seeded legume cover crops for no-tillage corn in the humid East. *Agron. J.* **82**, 117-124 (1990).

163 Holford, I. Effects of duration of grazed lucerne on long-term yields and nitrogen uptake of subsequent wheat. *Aust. J. Agric. Resour. Econ.* **31**, 239-250 (1980).

164 Holland, J.F. & Herridge, D.F. Production of summer crops in Northern New South Wales. II. Effects of tillage and crop rotation on yields of sorghum. *Aust. J. Agric. Resour. Econ.* **43**, 123-134 (1992).

165 Honeycutt, C.W., Clapham, W.M. & Leach, S.S. Crop rotation and N fertilization effects on growth, yield, and disease incidence in potato. *Am. J. Potato Res.* **73**, 45-61 (1996).

166 Hou, X.K., Zhai, R.C., Zhu, G.H. & Bei, L.X. Effects of crop rotation, continuous cropping and different cultivation methods on fertilizer utilization ratio of nitrogen and phosphorus. *J. Heilongjiang Aug.First Land Reclam Univ.*, 44-52 (1995).

167 Howard, D.D.W.T., Chambers, A.Y. & Lessman, G.M. Rotation and fertilization effects on corn and soybean yields and soybean cyst nematode populations in a no-tillage system. *Agron. J.* **90**, 518-522 (1998).

168 Hoyt, P.B. Residual effects of alfalfa and bromegrass cropping on yields of wheat grown for 15 subsequent years. *Can. J. Plant Sci* **70**, 109-113 (1990).

169 Huggins, D. R., Allmaras, R. R., Clapp, C. E., Lamb, J. A. & Randall, G. W. Corn-soybean sequence and tillage effects on soil carbon dynamics and storage. *Soil Sci. Soc. Am. J.* **71**, 145–154 (2007).

170 Ingver, A. et al. Leguminous pre-crops improved quality of organic winter and spring cereals. *Biol. Agric. Hortic* **35**, 46-60 (2019).

171 Iragavarapu, T.K.U.O., Randall, G.W. & Russelle, M.P. Yield and nitrogen uptake of rotated corn in a ridge tillage system. *Agron. J.* **89**, 397-403 (1997).

172 Izaurralde, R.C., Juma, N.G., McGill, W.B., Haderlein, L. & Choudhary, M. Crop and nitrogen yield in legume-based rotations practiced with zero tillage and low-input methods. *Agron. J.* **87**, 958-964 (1995).

Jain, N.K., Besarwal, H.S. & Dashora, L.N. Production potential, profitability, sustainability and energetics of different wheat (*Triticum aestivum*)-based cropping systems. *Arch. Agron. Soil Sci.* **57**, 477-487 (2011).

Janovicek, K.J.U.O., Vyn, T.J. & Voroney, R.P. No-till corn response to crop rotation and in-row residue placement. *Agron. J.* **89**, 588-596 (1997).

Jat, R.A., Dungrani, R.A., Arvadia, M.K. & Sahrawat, K.L. Diversification of rice (*Oryza Sativa* L.)-Based cropping systems for higher productivity, resource-use efficiency and economic returns in South Gujarat, India. *Arch. Agron. Soil Sci.* **58**, 561-572 (2012).

Jensen, C.R. et al. The effect of lupins as compared with peas and oats on the yield of the subsequent winter barley crop. *Eur. J. Agron.* **20**, 405-418 (2004).

Jeske, E.S., Tian, H., Hanford, K., Walters, D.T. & Drijber, R.A. Long-term nitrogen fertilization reduces extraradical biomass of arbuscular mycorrhizae in a maize (*zea mays* L.) cropping system. *Agric. Ecosyst. Environ.* **255**, 111-118 (2018).

Johnson, A.W., Dowler, C.C., Baker, S.H. & Handoo, Z.A. Crop yields and nematode population densities in triticale-cotton and triticale-soybean rotations. *J. Nematol.* **30**, 353 (1998).

Johnson, N. C., Copeland, P. J., Crookston, R. K. & Pfleger, F. L. Mycorrhizae: Possible explanation for yield decline with continuous corn and soybean. *Agron. J.* **84**, 387–390 (1992).

Jones, M. J. Effects of previous crop on yield and nitrogen response of maize at samaru, nigeria. *Exp. Agric.* **10**, 273–279 (1974).

Jones, M.J. & Singh, M. Time trends in crop yields in long-term trials. *Exp. Agric.* **36**, 165-179 (2000).

Karlen, D.L., Kovar, J.L., Cambardella, C.A. & Colvin, T.S. Thirty-year tillage effects on crop yield and soil fertility indicators. *Soil Tillage Res.* **130**, 24-41 (2013).

Katsvairo, T.W. & Cox, W.J. Tillage× rotation× management interactions in corn. *Agron. J.* **92**, 493-500 (2000).

Kaye, N.M., Mason, S.C., Galusha, T.D. & Mamo, M. Nodulating and non-nodulating soybean rotation influence on soil nitrate-nitrogen and water, and sorghum yield. *Agron. J.* **99**, 599-606 (2007).

Keisling. T. C., Scott. H.D., Waddle. B.A., Williams. W. & Frans. R.E. Winter cover crops influence on cotton yield and selected soil properties1. *Commun. Soil Sci. Plant Anal.* **25**, 3087–3100 (1994).

Kelley, K.W. & Sweeney, D.W. Placement of preplant liquid nitrogen and phosphorus fertilizer and nitrogen rate affects no-till wheat following different summer crops. *Agron. J.* **99**, 1009-1017 (2007).

Kermah, M. et al. Legume–maize rotation or relay? Options for ecological intensification of smallholder farms in the Guinea savanna of Northern Ghana. *Exp. Agric.* **55**, 673-691 (2019). Kermah, M. et al. Legume–maize rotation or relay? Options for ecological intensification of smallholder farms in the Guinea savanna of Northern Ghana. *Exp. Agric.* **55**, 673-691 (2020). Khakbazan, M., Gan, Y.T, Bandara, M. & Huang, J. Economics of pulse crop frequency and sequence in a wheat-based rotation. *Agron. J* **112**, 2058-2080 (2020).

Kimaro, A.A., Timmer, V.R., Chamshama, S.A.O., Ngaga, Y.N. & Kimaro, D.A. Competition
190 between maize and pigeonpea in semi-arid Tanzania: Effect on yields and nutrition of crops. *Agric. Ecosyst. Environ.* **134**, 115-125 (2009).

Kirkegaard, J.A. et al. Effect of previous crops on crown rot and yield of durum and bread
191 wheat in Northern NSW. *Aust. J. Agric. Resour. Econ.* **55**, 321-334 (2004).

Kouyaté, Z., Franzluebbers, K., Juo, A.S.R. & Hossner, L.R. Tillage, crop residue, legume
192 rotation, and green manure effects on sorghum and millet yields in the semiarid tropics of Mali. *Plant Soil* **225**, 141-151 (2000).

Kramberger, B., Gselman, A., Janzekovic, M., Kaligaric, M. & Bracko, B. Effects of cover
193 crops on soil mineral nitrogen and on the yield and nitrogen content of maize. *Eur. J. Agron.* **31**, 103-109 (2009).

Krupinsky, J. M., Tanaka, D. L., Lares, M. T. & Merrill, S. D. Leaf spot diseases of barley
194 and spring wheat as influenced by preceding crops. *Agron. J.* **96**, 259–266 (2004).

Krupinsky, J.M., Tanaka, D.L., Merrill, S.D., Liebig, M.A. & Hanson, J.D. Crop sequence
195 effects of 10 crops in the Northern Great Plains. *Agr. Syst.* **88**, 227-254 (2006).

Kumar Rao, J. V. D. K., Dart, P. J. & Sastry, P. V. S. S. Residual effect of pigeonpea
196 (*Cajanus cajan*) on yield and nitrogen response of maize. *Exp. Agric.* **19**, 131–141 (1983).

Kumar, K., Goh, K.M., Scott, W.R. & Frampton, C.M. Effects of N-15-labelled crop residues
197 and management practices on subsequent winter wheat yields, nitrogen benefits and recovery under field conditions. *J. Agric. Sci.* **136**, 35-53 (2001).

Kuo, S. & Jellum, E. J. Influence of winter cover crop and residue management on soil
198 nitrogen availability and corn. *Agron. J.* **94**, 501–508 (2002).

Kuo, S. & Jellum, E. J. Long-term winter cover cropping effects on corn (*Zea mays* L.)
199 production and soil nitrogen availability. *Biol. Fertil. Soils* **31**, 470–477 (2000).

Kureh, I., Kamara, A.Y. & Tarfa, B.D. Influence of cereal-legume rotation on striga control
200 and maize grain yield in farmers' fields in the Northern Guinea savanna of Nigeria. *J. Agri. Rural Develop. in the Tropics and Subtropics (JARTS)* **107**, 41-54 (2006).

Kutcher, H. R., Johnston, A. M., Bailey, K. L. & Malhi, S. S. Managing crop losses from
201 plant diseases with foliar fungicides, rotation and tillage on a Black Chernozem in Saskatchewan, Canada. *Field. Crop. Res.* **124**, 205–212 (2011).

Lamb, J.A., Dowdy, R.H., Anderson, J.L. & Allmaras, R.R. Water quality in an irrigated
202 sandy soil: Ridge tillage in rotated corn and soybean compared with full-width tillage in continuous corn. *Soil Tillage Res.* **48**, 167-177 (1998).

LaMondia, J.A. & Halbrendt, J.M. Rotation and green manure crops for management of lesion
203 and dagger nematodes. *J. Nematol.* **42**, 251-251 (2010).

Langdale, G. W., Wilson, R. L. & Bruce, R. R. Cropping frequencies to sustain long-term
204 conservation tillage systems. *Soil Sci. Soc. Am. J.* **54**, 193–198 (1990).

Lenssen, A.W., Johnson, G.D. & Carlson, G.R. Cropping sequence and tillage system 205
influences annual crop production and water use in semiarid Montana, USA. *Field. Crop. Res.* **100**, 32-43 (2007).

Li, Z.P., Wang, J., Shang, Y.Q. & Zhang, S.H. Decomposition of cover crop residues in soils
206 and its effects on winter wheat yield. *Agri. Res. Arid Areas* **37**, 75-82+90 (2019).

Liang, A. Lodging in corn varies with tillage and crop rotation: A case study after typhoon

207 bolaven pummeling over the black soil Zeon in Northeast China. *Pakistan Journal of Agricultural Sciences* **54**, 539-545 (2017).

208 Liben, F.M. et al. Conservation agriculture effects on crop productivity and soil properties in Ethiopia. *Agron. J.* **110**, 758-767 (2018).

209 Liben, F.M. et al. Conservation agriculture for maize and bean production in the Central Rift Valley of Ethiopia. *Agron. J.* **109**, 2988-2997 (2017).

210 Liebman, M., Drummond, F. A., Corson, S. & Zhang, J. Tillage and rotation crop effects on weed dynamics in potato production systems. *Agron. J.* **88**, 18–26 (1996).

211 Limon-Ortega, A., Govaerts, B. & Sayre, K.D. Straw management, crop rotation, and nitrogen source effect on wheat grain yield and nitrogen use efficiency. *Eur. J. Agron.* **29**, 21-28 (2008).

212 Lin, R. & Chen, C. Tillage, crop rotation, and nitrogen management strategies for wheat in Central Montana. *Agron. J.* **106**, 475-485 (2014).

213 Linh, T. B. *et al.* Inclusion of upland crops in rice-based rotations affects chemical properties of clay soil (vol 31, pg 313, 2015). *Soil Use Manag.* **31**, 544-544, doi:10.1111/sum.12218 (2015).

214 Linh, T.B., Sleutel, S., Vo Thi, G., Le Van, K. & Cornelis, W.M. Deeper tillage and root growth in annual rice-upland cropping systems result in improved rice yield and economic profit relative to rice monoculture. *Soil Tillage Res.* **154**, 44-52 (2015).

215 Liu, K., Bandara, M., Hamel, C., Knight, J.D. & Gan, Y.T. Intensifying crop rotations with pulse crops enhances system productivity and soil organic carbon in semi-arid environments. *Field Crop. Res.* **248**, 107657 (2020).

216 Lombin, L.G. Continuous cultivation and soil productivity in the semi-arid savannah: The influence of crop rotation. *Agron. J.* **73**, 357-363 (1981).

217 López-Fando, C. & Almendros, G. Interactive effects of tillage and crop rotations on yield and chemical properties of soils in semi-arid central Spain. *Soil Tillage Res.* **36**, 45–57 (1995).

218 Lund, M.G., Carter, P.R. & Oplinger, E.S. Tillage and crop rotation affect corn, soybean, and winter wheat yields. *J. Prod. Agric* **6**, 207-213 (1993).

219 Lupwayi, N.Z. & Soon, Y.K. Nitrogen-related rotational effects of legume crops on three consecutive subsequent crops. *Soil Sci. Soc. Am. J.* **80**, 306-316 (2016).

220 M. E. Probert, P. S. Carberry, R. L. McCown, & J. E. T. Nitrogen benefits of lupins, field pea, and chickpea to wheat production in south-eastern Australia. *Aust. J. Agric. Res.* **49**, 317–327 (1998).

221 Ma, B.L., Ying, J., Dwyer, L.M., Gregorich, E.G. & Morrison, M.J. Crop rotation and soil N amendment effects on maize production in Eastern Canada. *Can. J. Plant Sci.* **83**, 483-495 (2003).

222 Ma, B.L., Zheng, Z.M., Morrison, M.J. & Gregorich, E.G. Nitrogen and phosphorus nutrition and stoichiometry in the response of maize to various N rates under different rotation systems. *Nutr. Cycl. Agroecosystems* **104**, 93-105 (2016).

Maaz, T. & Pan, W. Residual fertilizer, crop sequence, and water availability impact rotational

223 nitrogen balances. *Agron. J.* **109**, 2839-2862 (2017).

224 Macholdt, J., Styczen, M.E., Macdonald, A., Piepho, H. & Honermeier, B. Long-term analysis from a cropping system perspective: Yield stability, environmental adaptability, and production risk of winter barley. *Eur. J. Agron.* **117**, 126056 (2020).

225 Mady Kaye, N., Mason, S.C., Jackson, D.S. & Galusha, T.D. Crop rotation and soil amendment alters sorghum grain quality. *Crop Sci.* **47**, 722-727 (2007).

226 Mahama, G.Y., Prasad, P.V.V., Roozeboom, K.L., Nippert, J.B. & Rice, C.W. Cover crops, fertilizer nitrogen rates, and economic return of grain sorghum. *Agron. J.* **108**, 1-16 (2016).

227 Mahler, R. L. & Auld, D. L. Evaluation of the green manure potential of Austrian winter peas in northern Idaho. *Agron. J.* **81**, 258–264 (1989).

228 Malik, R. S. *et al.* Dynamic crop sequencing in Western Australian cropping systems. *Crop Past. Sci.* **66**, 594–609 (2015).

229 Maloney, T.S., Silveira, K.G. & Oplinger, E.S. Rotational vs. Nitrogen-fixing influence of soybean on corn grain and silage yield and nitrogen use. *J. Prod. Agric.* **12**, 175-187 (1999).

230 Maltas, A., Corbeels, M., Scopel, E., Wery, J. & Macena da silva, F. A. Cover crop and nitrogen effects on maize productivity in no-tillage systems of the Brazilian cerrados. *Agron. J.* **101**, 1036–1046 (2009).

231 Mannan, M. A. Organic matter, nitrogen, and carbon: Nitrogen ratio of soils as affected by crops and cropping systems. *Soil Sci.* **93**, 83–86 (1961).

232 Marcelo, A.V., Corá, J.E., Fernandes, C., Martins, M.D.R. & Jorge, R.F. Crop sequences in no-tillage system: Effects on soil fertility and soybean, maize and rice yield. *Rev. Bras. Cienc. Solo* **33**, 417-428 (2009).

233 Martinrueda, I. et al. Tillage and crop rotation effects on barley yield and soil nutrients on a Calciortidic Haploxeralf. *Soil Tillage Res.* **92**, 1-9 (2007).

234 Mashingaidze, N., Twomlow, S., Madakadze, I.C., Mupangwa, W. & Mavunganidze, Z. Weed growth and crop yield responses to tillage and mulching under different crop rotation sequences in semi-arid conditions. *Soil Use Manage.* **33**, 311-327 (2017).

235 Masri, Z. & Ryan, J. Soil organic matter and related physical properties in a Mediterranean wheat-based rotation trial. *Soil Tillage Res.* **87**, 146–154 (2006).

236 Mazzoncini, M., Bàrberi, P., Cerrai, D., Rinaudo,V. & Belloni, P. Effects of green manure on soil nitrogen availability and crop productivity in a Mediterranean organic farming system. 1– 9 (2004).

237 Mbewe, D.N.M., Chishala, B.H., Chirwa, T.S. & Mafongoya, P.L. Changes in soil properties and their effects on maize productivity following *Sesbania Sesban* and *Cajanus Cajan* improved fallow systems in Eastern Zambia. *Biol. Fertil. Soils* **40**, 20-27 (2004).

238 McEwen, J., Darby, R.J., Hewitt, M.V. & Yeoman, D.P. Effects of field beans, fallow, lupins, oats, oilseed rape, peas, ryegrass, sunflowers and wheat on nitrogen residues in the soil and on the growth of a subsequent wheat crop. *J. Agric. Sci.* **115**, 209-219 (1990).

239 Meyer-Aurich, A., Janovicek, K., Deen, W. & Weersink, A. Impact of tillage and rotation on yield and economic performance in corn-based cropping systems. *Agron. J.* **98**, 1204–1212 (2006).

Milev, G., Tonev, T.K. & Kiryakova, V. Influence of some agronomy factors on spike

240 components after rare incidence of fusarium head blight epiphytoty of winter wheat. II. Effect of post-harvest residue incorporation. *Bulg. J. Agric. Sci* **14**, 41 (2008).

Mohammed, M.S. & Clegg, M.D. Pearl millet-soybean rotation and nitrogen fertilizer effects
241 on millet productivity. *Agron. J* **85**, 1009-1013 (1993).

Montemurro, F. & Maiorana, M. Agronomic practices at low environmental impact for durum
242 wheat in Mediterranean conditions. *J. Plant Nutr.* **38**, 624-638 (2015).

Montemurro, F. & Maiorana, M. Cropping systems, tillage and fertilization strategies for
243 durum wheat performance and soil properties. *Int. J. Plant Prod.* **8**, 51-75 (2014).

Moyer, J.R., Blackshaw, R.E., Doram, R.C., Huang, H.C. & Entz, T. Effect of previous crop
244 and herbicides on weed growth and wheat yield. *Can. J. Plant Sci.* **85**, 735-746 (2005).

Munyinda, K., O'Halloran, I.P. & Karamanos, R.E. Yields of wheat in rotation with maize and
245 soybeans in Zambia. *Can. J. Plant Sci.* **68**, 747-753 (1988).

Muoni, T., Mhlanga, B., Forkman, J., Sitali, M. & Thierfelder, C. Tillage and crop rotations
246 enhance populations of earthworms, termites, dung beetles and centipedes: Evidence from a long-term trial in Zambia. *J. Agric. Sci.* **157**, 504-514 (2019).

Mupangwa, W., Thierfelder, C. & Ngwira, A. Fertilization strategies in conservation 247
agriculture systems with maize–legume cover crop rotations in Southern Africa. *Exp. Agric.* **53**, 288-307 (2017).

Mussgnug, F., Becker, M., Son, T.T., Buresh, R.J. & Vlek, P.L.G. Yield gaps and nutrient
248 balances in intensive, rice-based cropping systems on degraded soils in the Red River Delta of Vietnam. *Field. Crop. Res.* **98**, 127-140 (2006).

Naab, J.B., Mahama, G.Y., Yahaya, I. & Prasad, P.V.V. Conservation agriculture improves
249 soil quality, crop yield, and incomes of smallholder farmers in North Western Ghana. *Front. Plant Sci.* **8**, 996 (2017).

Nafziger, E.D. & Dunker, R.E. Soil organic carbon trends over 100 years in the Morrow Plots.
250
Agron. J **103**, 261-267 (2011).

Ncube, B., Dimes, J.P., van Wijk, M.T., Twomlow, S.J. & Giller, K.E. Productivity and residual benefits of grain legumes to sorghum under semi-arid conditions in South-western
251
Zimbabwe: Unravelling the effects of water and nitrogen using a simulation model. *Field. Crop. Res.* **110**, 173-184 (2009).

Ngwira, A.R., Kabambe, V., Simwaka, P., Makoko, K. & Kamoyo, K. Productivity and
252 profitability of maize-legume cropping systems under conservation agriculture among smallholder farmers in Malawi. *Acta Agric. Scand B Soil Plant Sci.* **70**, 241-251 (2020).

Nielsen, D. C. & Vigil, M. F. Legume green fallow effect on soil water content at wheat
253 planting and wheat yield. *Agron. J.* **97**, 684–689 (2005).

Niu, Y., Bainard, L., Bandara, M.S., Hamel, C. & Gan, Y. Soil residual water and nutrients
254 explain about 30% of the rotational effect in 4-year pulse-intensified rotation systems. *Can. J. Plant Sci.* **97**,852-864 (2017).

Norwood, C.A. Dryland winter wheat as affected by previous crops. *Agron. J* **92**, 121-127
255 (2000).

256 Nouri, A. et al. Crop species in no-tillage summer crop rotations affect soil quality and yield in an Alfisol. *Geoderma* **345**, 51-62 (2019).

257 Nyagumbo, I. et al. Maize yield effects of conservation agriculture based maize–legume cropping systems in contrasting agro-ecologies of Malawi and Mozambique. *Nutr. Cycl. Agroecosystems* **105**, 275-290 (2016).

258 Nykänen, A., Granstedt, A. & Jauhiainen, L. Residual effect of clover-rich leys on soil nitrogen and successive grain crops. *AFSci* **17**, 73-87 (2008).

259 O'Donovan, J. T. *et al.* Effect of preceding crop and nitrogen application on malting barley quality. *Can. J. Plant Sci.* **97**, 1014–1023 (2017).

260 O'donovan, J. T. *et al.* Rotational effects of legumes and non-legumes on hybrid canola and malting barley. *Agron. J.* **106**, 1921–1932 (2014).

261 Obour, A.K., Stahlman, P.W. & Holman, J.D. Soil chemical properties as influenced by long term glyphosate-resistant corn and soybean production in the central Great Plains, USA. *Geoderma* **277**, 1-9 (2016).

262 Oikeh, S.O. et al. Integrated soil fertility management involving promiscuous dual-purpose soybean and upland NERICA enhanced rice productivity in the savannas. *Nutr. Cycl. Agroecosystems* **88**, 29-38 (2010).

263 Oikeh, S.O. et al. Integrated soil fertility management involving promiscuous dual-purpose soybean and upland nerica enhanced rice productivity in the savannas. *Nutr. Cycl. Agroecosystems* **88**, 29-38 (2011).

264 Ojiem, J.O., Franke, A.C., Vanlauwe, B., de Ridder, N. & Giller, K.E. Benefits of legume–maize rotations: Assessing the impact of diversity on the productivity of smallholders in Western Kenya. *Field. Crop. Res.* **168**, 75-85 (2014).

265 Oliveira, M., Castro, C., Coutinho, J. & Trindade, H. N supply and pre-cropping benefits to triticale from three legumes in rainfed and irrigated Mediterranean crop rotations. *Field. Crop. Res.* **237**, 32-42 (2019).

266 Oswald, A. & Ransom, J.K. Striga control and improved farm productivity using crop rotation. *Crop Prot.* **20**, 113-120 (2001).

267 Owen, K.J., Clewett, T.G., Bell, K.L. & Thompson, J.P. Wheat biomass and yield increased when populations of the root-lesion nematode (*Pratylenchus thornei*) were reduced through sequential rotation of partially resistant winter and summer crops. *Crop Past. Sci.* **65**, 227 (2014).

268 Oyer, L.J. & Touchton, J.T. Utilizing legume cropping systems to reduce nitrogen fertilizer requirements for conservation-tilled corn. *Agron. J.* **82**, 1123-1127 (1990).

269 Pal, M.S. Alternative crop production strategies for rice-wheat cropping systems in the Indo-Gangetic Plains of India. *Aust. J. Exp. Agric.* **43**, 605 (2003).

270 Papastylianou, I. Effect of preceding legume or cereal on barley grain and nitrogen yield. *J. Agric. Sci.* **108**, 623–626 (1987).

271 Papastylianou, I. Productivity and nitrogen fertilizer requirements of barley in rotation systems in rainfed Mediterranean conditions. *Eur. J. Agron.* **2**, 119–129 (1993).

272 Paré, T., Chalifour, F.P., Bourassa, J. & Antoun, H. Forage-corn production and N fertilizer replacement values following 1 or 2 years of legumes. *Can. J. Plant Sci.***73**, 477-493 (1993).

Parsons, K.J., Zheljazkov, V.D., MacLeod, J. & Caldwell, C.D. Soil and tissue phosphorus,

273 potassium, calcium, and sulfur as affected by dairy manure application in a no-till corn, wheat, and soybean rotation. *Agron. J.* **99**, 1306-1316 (2007).

Partey, S.T., Saito, K., Preziosi, R.F. & Robson, G.D. Biochar use in a legume-rice rotation
274 system: Effects on soil fertility and crop performance. *Arch. Agron. Soil Sci.* **62**, 199-215 (2016).

Perez-Brandan, C. *et al.* The effect of crop sequences on soil microbial, chemical and physical
275 indicators and its relationship with soybean sudden death syndrome (complex of Fusarium species). *Span J. Agric. Res.* **12**, 252–264 (2014).

276 Peterson, T. A. & Varvel, G. E. Crop yield as affected by rotation and nitrogen rate. III. Corn. *Agron. J.* **81**, 735–738 (1989).

Peterson, T.A. & Varvel, G.E. Crop yield as affected by rotation and nitrogen rate. II. Grain
277 sorghum. *Agron. J.* **81**, 731-734 (1989).

Pikul Jr, J.L., Osborne, S.L. & Riedell, W.E. Corn yield and nitrogen-and water-use under no-
278 tillage rotations. *Commun. Soil Sci. Plant Anal.* **43**, 2722-2734 (2012).

Pikul, J. L., Aase, J. K. & Cochran, V. L. Lentil green manure as fallow replacement in the
279 semiarid northern Great Plains. *Agron. J.* **89**, 867–874 (1997).

Pikul, J. L., Hammack, L. & Riedell, W. E. Corn yield, nitrogen use, and corn rootworm
280 infestation of rotations in the northern corn belt. *Agron. J.* **97**, 854–863 (2005).

Porter, P. M., Crookston, R. K., Ford, J. H., Huggins, D. R. & Lueschen, W. E. Interrupting
281 yield depression in monoculture corn: Comparative effectiveness of grasses and dicots. *Agron. J.* **89**, 247–250 (1997).

Potratz, D.J. et al. Strip-till, other management strategies, and their interactive effects on corn
282 grain and soybean seed yield. *Agron. J* **112**, 72-80 (2020).

Prew, R. D. & Dyke, G. V. Experiments comparing 'break crops' as a preparation for winter
283 wheat followed by spring barley. *J. Agric. Sci.* **92**, 189–201 (1979).

284 Propheter, J.L. & Staggenborg, S. Performance of annual and perennial biofuel crops: Nutrient removal during the first two years. *Agron. J.* **102**, 798-805 (2010).

285 Propheter, J.L. & Staggenborg, S. Performance of annual and perennial biofuel crops: Nutrient removal during the first two years. *Agron. J.* **102**, 798-805 (2011).

Przednowek, D., Entz, M.H., Irvine, B., Flaten, D.N. & Thiessen Martens, J.R. Rotational 286
yield and apparent N benefits of grain legumes in Southern Manitoba. *Can. J. Plant Sci.***84**, 1093-1096 (2004).

Qin, W., Zhang, X., Chen, S., Sun, H. & Shao, L. Crop rotation and N application rate 287
affecting the performance of winter wheat under deficit irrigation. *Agric. Water Manag.* **210**, 330-339 (2018).

Raimbault, B.A. & Vyn, T.J. Crop rotation and tillage effects on corn growth and soil
288 structural stability. *Agron. J.* **83**, 979-985 (1991).

289 Rao, M.R. & Mathuva, M.N. Legumes for improving maize yields and income in semi-arid Kenya. *Agric. Ecosyst. Environ.* **78**, 123-137 (2000).

Rasiah, V. Temporal dynamics of soil nitrate-n after termination of forage phases in corn-
290 forage rotation. *Biol. Fertil. Soils* **29**, 201-206 (1999).

Reddy, K. N., Zablotowicz, R. M. & Krutz, L. J. Corn and soybean rotation under reduced tillage management: Impacts on soil properties, yield, and net return. *Am. J. Plant Sci.* **04**, 10– 17 (2013).

Rego, T. J. & Nageswara Rao, V. Long-term effects of grain legumes on rainy-season sorghum productivity in a semi-arid tropical Vertisol. *Exp. Agric.* **36**, 205–221 (2000).

Rembon, F.S. & MacKenzie, A.F. Soybean nitrogen contribution to corn and residual nitrate under conventional tillage and no-till. *Can. J. Plant Sci.* **77**, 543-551 (1997).

Rice, W. A., Olsen, P. E., Bailey, L. D., Biederbeck, V. O. & Slinkard, A. E. The use of annual legume green-manure crops as a substitute for summerfallow in the Peace River region. *Can. J. Soil Sci.* **73**, 243–252 (1993).

Riedell, W.E. et al. Corn and soil fertility responses to crop rotation with low, medium, or high inputs. *Crop Sci.* **38**, 427-433 (1998).

Rochester, I.J., Peoples, M.B., Hulugalle, N.R., Gault, R.R. & Constable, G.A. Using legumes to enhance nitrogen fertility and improve soil condition in cotton cropping systems. *Field. Crop. Res.* **70**, 27-41 (2001).

Roder, W., Mason, S. C., Clegg, M. D. & Kniep, K. R. Crop root distribution as infl uenced by grain sorghum–soybean rotation and fertilization. *Soil Sci. Soc. Am. J.* **53**, 1464–1470 (1989).

Roder, W., Mason, S.C., Clegg, M.D. & Kniep, K.R. Yield-soil water relationships in sorghum-soybean cropping systems with different fertilizer regimes. *Agron. J.* **81**, 470-475 (1989).

Rogers, C. W., Smartt, A. D., Brye, K. R. & Norman, R. J. Nitrogen source effects on methane emissions from drill-seeded, delayed-flood rice production. *Soil Sci.* **182**, 9–17 (2017).

Ross, S.M. et al. The effects of three pulse crops on a second subsequent crop. *Can. J. Plant Sci.* **95**, 779-786 (2015).

Rubin, J.C., Struffert, A.M., Fernández, F.G. & Lamb, J.A. Maize yield and nitrogen use efficiency in upper Midwest irrigated sandy soils. *Agron. J.* **108**, 1681-1691 (2016).

Ryan, J. et al. Crop sequences, nitrogen fertilizer and grazing intensity in relation to wheat yields in rainfed systems. *J. Agric. Sci.* **148**, 205-216 (2010).

Sadras, V.O., Baldock, J.A., Cox, J.W. & Bellotti, W.D. Crop rotation effect on wheat grain yield as mediated by changes in the degree of water and nitrogen co-limitation. *Aust J Agric Resour Econ* **55**, 599-607 (2004).

Sainju, U. M., Lenssen, A. W., Caesar-TonThat, T. & Evans, R. G. Dryland crop yields and soil organic matter as influenced by long-term tillage and cropping sequence. *Agron. J.* **101**, 243–251 (2009).

Sainju, U. M., Singh, B. P., Whitehead, W. F. & Wang, S. Carbon supply and storage in tilled and nontilled soils as influenced by cover crops and nitrogen fertilization. *J. Environ. Qual.* **35**, 1507–1517 (2006).

Sainju, U. M., Whitehead, W. F. & Singh, B. P. Biculture legume-cereal cover crops for enhanced biomass yield and carbon and nitrogen. *Agron. J.* **97**, 1403–1412 (2005).

Sainju, U.M. Cropping sequence and nitrogen fertilization impact on surface residue, soil

carbon sequestration, and crop yields. *Agron. J.* **106**, 1231-1242 (2015).

Sainju, U.M., Lenssen, A.W. & Barsotti, J.L. Dryland malt barley yield and quality affected by tillage, cropping sequence, and nitrogen fertilization. *Agron. J.* **105**, 329-340 (2013).

Sainju, U.M., Stevens, W.B., Caesar-TonThat, T. & Liebig, M.A. Soil greenhouse gas emissions affected by irrigation, tillage, crop rotation, and nitrogen fertilization. *J. Environ. Qual.* **41**, 1774-1786 (2012).

Salim Khan, M. A., Katti, M. S. & Raja, V. Fodder legumes affecting sequential crop production and fertilizer N use efficiency. *J. Agric. Sci.* **105**, 1–7 (1985).

Salmerón, M., Cavero, J., Quílez, D. & Isla, R. Winter cover crops affect monoculture maize yield and nitrogen leaching under irrigated Mediterranean conditions. *Agron. J.* **102**, 1700– 1709 (2010).

Salmerón, M., Isla, R. & Cavero, J. Effect of winter cover crop species and planting methods on maize yield and N availability under irrigated Mediterranean conditions. *Field. Crop. Res.* **123**, 89-99 (2011).

Sanford, J.O. & Hairston, J.E. Effects of N fertilization on yield, growth, and extraction of water by wheat following soybeans and grain sorghum. *Agron. J.* **76**, 623-627 (1984).

Schahbazian, N. & Gretzmacher, R. Improving the productivity of winter wheat in Iran through rotation of wheat, fallow, soybean and alfalfa and manuring. *Bodenkultur* **49**, 151-157 (1998).

Schlegel, A.J. et al. Limited irrigation of corn-based no-till crop rotations in West Central Great Plains. *Agron. J.* **108**, 1132-1141 (2016).

Schlegel, A.J., Assefa, Y., Haag, L.A., Thompson, C.R. & Stone, L.R. Soil water and water use in long-term dryland crop rotations. *Agron. J.* **111**, 2590-2599 (2019).

Schlegel, A.J., Assefa, Y., Haag, L.A., Thompson, C.R. & Stone, L.R. Yield and overall productivity under long-term wheat-based crop rotations: 2000 through 2016. *Agron. J.* **111**, 264-274 (2019).

Schulz, S., Keatinge, J. & Wells, G.J. Productivity and residual effects of legumes in rice-based cropping systems in a warm-temperate environment: I. Legume biomass production and N fixation. *Field. Crop. Res.* **61**, 23-35 (1999).

Schulz, S., Keatinge, J.D.H., J. Wells, G. & Shrestha, R. Effect of legume management on forage production and residual effects on upland rice. *J. Agron. Crop Sci.* **184**, 173-180 (2000).

Schwenke, G. D., Brock, P. M., Haigh, B. M. & Herridge, D. F. Greenhouse gas emission reductions in subtropical cereal-based cropping sequences using legumes, DMPP-coated urea and split timings of urea application. *Soil Res.* **56**, 724-736, doi:10.1071/sr18108 (2018).

Shafi, M., Bakht, J., & Khan, M.A. Effect of crop sequence and crop residues on soil C, soil N and yield of maize. *Pakistan Journal of Botany* **42**, 1651-1664 (2010).

Shafi, M., Bakht, J., Jan, M. & Shah, Z. Soil C and N dynamics and maize (*Zea may* l.) Yield as affected by cropping systems and residue management in North-western Pakistan. *Soil Tillage Res.* **94**, 520-529 (2007).

Shah, Z., Shah, S.H., Peoples, M.B., Schwenke, G.D. & Herridge, D.F. Crop residue and fertiliser N effects on nitrogen fixation and yields of legume–cereal rotations and soil organic fertility. *Field. Crop. Res.* **83**, 1-11 (2003).

Shahzad, M., Farooq, M., Jabran, K. & Hussain, M. Impact of different crop rotations and tillage systems on weed infestation and productivity of bread wheat. *Crop Prot.* **89**, 161-169 (2016).

Sharma, A. R. & Behera, U. K. Nitrogen contribution through Sesbania green manure and dual-purpose legumes in maize-wheat cropping system: Agronomic and economic considerations. *Plant Soil* **325**, 289–304 (2009).

Sharma, A.R. & Behera, U.K. Recycling of legume residues for nitrogen economy and higher productivity in maize (*zea mays*)–wheat (*Triticum Aestivum*) cropping system. *Nutr. Cycl. Agroecosystems* **83**, 197-210 (2009).

Sharma, S. N. & Prasad, R. Effects of Sesbania green manuring and mungbean residue incorporation of productivity and nitrogen uptake of a rice-wheat cropping system. *Bioresour. Technol.* **67**, 171–175 (1999).

Sharma, S.N.I.A., Prasad, R. & Singh, S. Residual effects of growing mungbean and uridbean on the yield and nitrogen uptake of a succeeding wheat crop. *Fertil. Res.* **44**, 163-168 (1996).

Sheaffer, C.C., Simmons, S.R. & Schmitt, M.A. Annual medic and berseem clover dry matter and nitrogen production in rotation with corn. *Agron. J.* **93**, 1080-1086 (2001).

Siadat, S. A. *et al.* Rapeseed *(Brassica napus* L. var. oleifera) response to nitrogen fertilizer following different previous crops. *Ital. J. Agron.* **6**, 199–203 (2011).

Sieling, K. & Christen, O. Crop rotation effects on yield of oilseed rape, wheat and barley and residual effects on the subsequent wheat. *Arch. Agron. Soil Sci.* **61**, 1531-1549 (2015).

Sieling, K., Christen, O., Nemati, B. & Hanus, H. Effects of previous cropping on seed yield and yield components of oil-seed rape (*Brassica napus* L.). *Eur. J. Agron.* **6**, 215–223 (1997). Sileshi, G. & Mafongoya, P.L. Effect of rotational fallows on abundance of soil insects and weeds in maize crops in Eastern Zambia. *Appl. Soil Ecol.* **23**, 211-222 (2003).

Sincik, M., Turan, Z. M. & Göksoy, A. T. Responses of potato (*Solanum tuberosum* L.) to green manure cover crops and nitrogen fertilization rates. *Am. J. Potato Res.* **85**, 150–158 (2008).

Sindelar, A.J., Schmer, M.R., Jin, V.L., Wienhold, B.J. & Varvel, G.E. Crop rotation affects corn, grain sorghum, and soybean yields and nitrogen recovery. *Agron. J.* **108**, 1592-1602 (2016).

Sindelar, A.J., Schmer, M.R., Jin, V.L., Wienhold, B.J. & Varvel, G.E. Long-term corn and soybean response to crop rotation and tillage. *Agron. J.* **107**, 2241-2252 (2015).

Singer, J. W. & Cox, W. J. Corn growth and yield under different crop rotation, tillage, and management systems. *Crop Sci.* **38**, 996–1003 (1998).

Singer, J.W. & Cox, W.J. Agronomics of corn production under different crop rotations in New York. *J. Prod. Agric* **11**, 462-468 (1998).

Singh, R.K., Bohra, J.S., Nath, T., Singh, Y. & Singh, K. Integrated assessment of diversification of rice-wheat cropping system in Indo-Gangetic plain. *Arch. Agron. Soil Sci.* **57**, 489-506 (2011).

Singh, S., Prasad, R., Singh, B. V., Goyal, S. K. & Sharma, S. N. Effect of green manuring, blue-green algae and neem-cake-coated urea on wetland rice (*Oryza sativa* L.). *Biol. Fertil. Soils* **9**, 235–238 (1990).

341 Singh, V.K., Sharma, B.B. & Dwivedi, B.S. The impact of diversification of a rice–wheat cropping system on crop productivity and soil fertility. *J. Agric. Sci.* **139**, 405-412 (2002).

342 Slope, D. B. & Etheridge, J. Effects of previous cropping and N fertilizer on grain yield and take-all in spring barley. *J. Agric. Sci.* **89**, 459–465 (1977).

343 Smiley, R. W., Ingham, R. E., Uddin, W. & Cook, G. H. Crop sequences for managing cereal cyst nematode and fungal pathogens of winter wheat. *Plant Dis.* **78**, 1142–1149 (1994).

344 Smith, B.J., Kirkegaard, J.A. & Howe, G.N. Impacts of brassica break-crops on soil biology and yield of following wheat crops. *Aust. J. Agric. Resour. Econ.* **55**, 1-11 (2004).

345 Smith, R.G. & Gross, K.L. Weed community and corn yield variability in diverse management systems. *Weed Sci.* **54**, 106-113 (2006).

346 Soon, Y.K. & Arshad, M.A. Contribution of di-nitrogen fixation by pea to the productivity and N budget of a wheat-based cropping system. *J. Agric. Sci.* **142**, 629-637 (2004).

347 Soon, Y.K. & Darwent, A.L. Effect of integrated management of couch grass (*Elytrigia repens*) on soil quality and crop nutrition. *J. Agric. Sci.* **130**, 323-328 (1998).

348 Sparrow, S.D., Cochran, V.L. & Sparrow, E.B. Residual effects of harvested and green-manured legumes on a subsequent barley crop in a subarctic environment. *Can. J. Plant Sci.***75**, 453-456 (1995).

349 St. Luce, M. et al. Legumes can reduce economic optimum nitrogen rates and increase yields in a wheat–canola cropping sequence in Western Canada. *Field. Crop. Res.* **179**, 12-25 (2015).

350 Stanger, T.F. & Lauer, J.G. Corn grain yield response to crop rotation and nitrogen over 35 years. *Agron. J.* **100**, 643-650 (2008).

351 Starovoytov, A., Gallagher, R. S., Jacobsen, K. L., Kaye, J. P. & Bradley, B. Management of small grain residues to retain legume-derived nitrogen in corn cropping systems. *Agron. J.* **102**, 895–903 (2010).

352 Stecker, J.A., Buchholz, D.D., Hanson, R.G., Wollenhaupt, N.C. & McVay, K.A. Tillage and rotation effects on corn yield response to fertilizer nitrogen on Aqualf soils. *Agron. J.* **87**, 409415 (1995).

353 Stempkowski, L. A. *et al.* Management of wheat stripe mosaic virus by crop rotation. *Eur. J. Plant Pathol.* **158**, 349-361, doi:10.1007/s10658-020-02077-8 (2020).

354 Stevens, W.B., Sainju, U.M., Caesar-TonThat, T. & Iversen, W.M. Malt barley yield and quality affected by irrigation, tillage, crop rotation, and nitrogen fertilization. *Agron. J.* **107**, 2107-2119 (2015).

355 Strom, N., Hu, W., Haarith, D., Chen, S. & Bushley, K. Interactions between soil properties, fungal communities, the soybean cyst nematode, and crop yield under continuous corn and soybean monoculture. *Appl. Soil Ecol.* **147**, 103388 (2020).

356 Strong, W. M., Harbison, J., Nielsen, R. G. H., Hall, B. D. & Best, E. K. Nitrogen availability in a darling downs soil following cereal, oilseed and grain legume crops. 1. Soil nitrogen accumulation. *Aust. J. Exp. Agric.* **26**, 347–351 (1986).

357 Stute, J.K. & Posner, J.L. Legume cover crops as a nitrogen source for corn in an oat-corn rotation. *J. Prod. Agric.* **8**, 385-390 (1995).

358 Sun, T. & Li, Z. Alfalfa-corn rotation and row placement affects yield, water use, and economic returns in Northeast China. *Field. Crop. Res.* **241**, 107558 (2019).

Swanepoel, C.M. et al. The benefits of conservation agriculture on soil organic carbon and

359 yield in Southern Africa are site-specific. *Soil Tillage Res.* **183**, 72-82 (2018).

360 Swanton, C.J., Chandler, K. & Janovicek, K.J. Integration of cover crops into no-till and ridge-till wheat (*triticum aestivum* L.)–corn (*Zea mays* L.) cropping sequence. *Can. J. Plant Sci.***76**, 85-91 (1996).

361 Sweeney, D. W. & Moyer, J. L. Legume green manures and conservation tillage for grain sorghum production on prairie soil. *Soil Sci. Soc. Am. J.* **58**, 1518–1524 (1994).

362 Szymanska, G. et al. The long-term effect of legumes as forecrops on the productivity of rotation winter triticale-winter rape with nitrogen fertilisation. *Acta Agric. Scand B Soil Plant Sci.* **70**, 128-134 (2020).

363 Tan, C.S., Drury, C.F., Reynolds, W.D., Groenevelt, P.H. & Dadfar, H. Water and nitrate loss through tiles under a clay loam soil in Ontario after 42 years of consistent fertilization and crop rotation. *Agric. Ecosyst. Environ.* **93**, 121-130 (2002).

364 Tanaka, D. L., Krupinsky, J. M., Merrill, S. D., Liebig, M. A. & Hanson, J. D. Dynamic cropping systems for sustainable crop production in the northern Great Plains. *Agron. J.* **99**, 904–911 (2007).

365 Tanaka, D. L., Liebig, M. A., Krupinsky, J. M. & Merrill, S. D. Crop sequence influences on sustainable spring wheat production in the northern Great Plains. *Sustainability* **2**, 3695–3709 (2010).

366 Taner, A., Kaya, Y., Aisoy, R.Z., Gültekin, 0. & Partigöç, F. Effect of tillage systems on energy use efficiency in wheat based cropping sequence. *Int. J. Agric. Biol.* **18**, 353-361 (2016).

367 Tang, W.G. et al. Impacts of winter planting patterns on soil heavy metal content and grain quality in late rice in double cropping rice area. *Acta Agron. Sin.* **37**, 1457-1464 (2011).

368 Tedla, A., Mamo, T., Klaij, M.C. & Diedhiou, M.L. Effects of cropping system, seed bed management and fertility interactions on biomass of crops grown on a Vertisol in the central highlands of Ethiopia. *J. Agron. Crop. Sci.* **183**, 205-211 (1999).

369 Tekin, S. et al. Comparison of wheat-based rotation systems and monocropping systems under dryland Mediterranean conditions. *Int J Agr Biol Eng* **10,** 203-213 (2017).

370 Thierfelder, C. & Wall, P.C. Rotation in conservation agriculture systems of Zambia: Effects on soil quality and water relations. *Exp. Agric.* **46**, 309-325 (2010).

371 Thomas, G.A. et al. Crop rotations for sustainable grain production on a Vertisol in the semi-arid subtropics. *J. Sustain. Agric.* **35**, 2-26 (2010).

372 Thomas, G.A. et al. Pasture - crop rotations for sustainable production in a wheat and sheep-based farming system on a Vertosol in South-west Queensland, Australia. *Anim. Prod. Sci.* **49**, 682 (2009).

373 Tian, G., Kolawole, G. O., Kang, B. T. & Kirchhof, G. Nitrogen fertilizer replacement indexes of legume cover crops in the derived savanna of West Africa. *Plant Soil* **224**, 287–296 (2000).

374 Toomsan, B. et al. Biological N-2 fixation and residual N benefit of pre-rice leguminous crops and green manures. *NJAS-WAGEN J LIFE SC* **48**, 19-29 (2000).

375 Toomsan, B., McDonagh, J. F., Limpinuntana, V. & Giller, K. E. Nitrogen fixation by groundnut and soyabean and residual nitrogen benefits to rice in farmers' fields in Northeast Thailand. *Plant Soil* **175**, 45–56 (1995).

376 Torbert, H.A., Reeves, D.W. & Mulvaney, R.L. Winter legume cover crop benefits to corn: Rotation vs. Fixed-nitrogen effects. *Agron. J.* **88**, 527-535 (1996).

Torstensson, G. Nitrogen delivery and utilization by subsequent crops after incorporation of leys with different plant composition. *Biol. Agric. Hortic* **16**, 129-143 (1998).

Tounkara, A. et al. Inorganic fertilizer use efficiency of millet crop increased with organic fertilizer application in rainfed agriculture on smallholdings in Central Senegal. *Agric. Ecosyst. Environ.* **294**, 106878 (2020).

Traore, H., Barro, A., Yonli, D., Stewart, Z. & Prasad, V. Water conservation methods and cropping systems for increased productivity and economic resilience in Burkina Faso. *Water* **12**, 976 (2020).

Turco, R.F., Bischoff, M., Breakwell, D.P. & Griffith, D.R. Contribution of soil-borne bacteria to the rotation effect in corn. *Plant Soil* **122**, 115-120 (1990).

Van Duijnen, R., Roy, J., Härdtle, W. & Temperton, V.M. Precrop functional group identity affects yield of winter barley but less so high carbon amendments in a mesocosm experiment. *Front. Plant Sci.* **9**,912 (2018).

Vanlauwe, B. et al. Utilization of rock phosphate by crops on a representative toposequence in the Northern Guinea savanna zone of Nigeria: Response by maize to previous herbaceous legume cropping and rock phosphate treatments. *Soil Biol. Biochem.* **32**, 2079-2090 (2000).

Vanotti, M. B. & Bundy, L. G. Soybean effects on soil nitrogen availability in crop rotations. *Agron. J.* **87**, 676–680 (1995).

Varvel, G.E. & Peterson, T.A. Nitrogen fertilizer recovery by corn in monoculture and rotation systems. *Agron. J.* **82**, 935-938 (1990).

Varvel, G.E. & Peterson, T.A. Nitrogen fertilizer recovery by grain sorghum in monoculture and rotation systems. *Agron. J.* **83**, 617-622 (1991).

Varvel, G.E. & Wilhelm, W.W. Soybean nitrogen contribution to corn and sorghum in Western corn belt rotations. *Agron. J.* **95**, 1220-1225 (2003).

Varvel, G.E. Monoculture and rotation system effects on precipitation use efficiency of corn. *Agron. J.* **86**, 204-208 (1994).

Varvel, G.E. Precipitation use efficiency of soybean and grain sorghum in monoculture and rotation. *Soil Sci. Soc. Am. J.* **59**, 527-531 (1995).

Varvel, G.E., Klocke, N.L. & Wilhelm, W. Corn-soybean rotation effects on soil and plant N indices. *Amer. Soc. Agri. Eng.* **95**, 235-238 (1995).

Veloso, M. G., Cecagno, D. & Bayer, C. Legume cover crops under no-tillage favor organomineral association in microaggregates and soil C accumulation. *Soil Tillage Res.* **190**, 139–146 (2019).

Videnovic, Z. et al. Effect of long term crop rotation and fertiliser application on maize productivity. *Turk. J. Field Crops* **18**, 233-237 (2013).

Vogel, A. & Below, F. Hybrid selection and agronomic management to lessen the continuous corn yield penalty. *Agronomy* **8**, 228 (2018).

Vogel, A.M. & Below, F.E. Residue and agronomic management to reduce the continuous corn yield penalty. *Agronomy (Basel)* **9**, 567 (2019).

394 Vrtílek, P., Smutný, V., Dryšlová, T., Neudert, L. & Křen, J. The effect of agronomic measures on grain yield of winter wheat in drier conditions. *Plant Soil Environ.* **65**, 63-70 (2019).

395 Wagger, M. G. Cover Cover crops management and nitrogen rate in relation to growth and yield of no-till corn. *Agron. J.* **81**, 533–538 (1989).

396 Wagger, M.G. & Denton, H.P. Crop and tillage rotations: grain yield, residue cover, and soil water. *Soil Sci. Soc. Am. J.* **56**, 1233-1237 (1992).

397 Wang, L. et al. Effects of seven diversified crop rotations on selected soil health indicators and wheat productivity. *Agronomy* **10**, 235 (2020).

398 Wang, S.B. et al. Effects of multiple winter cropping on soil organic carbon and double-cropping rice yield in the south of China. *Acta Ecol. Sin.* **38**, 6603-6610 (2018).

399 Wang, Y.M., Lei, Y.F., Wei, Z.W. & Han, Z.S. Effects of different rotation modes on yield, quality of silage corn, and soil fertility. *Acta Agric. Nucl. Sin.* **31**, 1803-1810 (2017).

400 Warman, P. R. Effect of incorporated green manure crops on subsequent oat production in an acid, infertile silt loam. *Plant Soil* **134**, 115–119 (1991).

401 Watkins, K.B., Anders, M.M. & Windham, T.E. An economic comparison of alternative rice production systems in Arkansas. *J. Sustain. Agric.* **24**, 57-78 (2004).

402 Watson, E. R. The influence of subterranean clover pastures on soil fertility i. Short-term effects. *Aust. J. Agric. Res.* **14**, 796–807 (1963).

403 Watson, E. R., Lapins, P. & Barron, R. J. W. Effects of initial clover seeding rate and length of ley on pasture production, soil nitrogen and crop yields in a ley farming system. *Aust. J. Exp. Agric.* **16**, 484–490 (1976).

404 Wei, X., Hao, M., Shao, M. & Gale, W. J. Changes in soil properties and the availability of soil micronutrients after 18 years of cropping and fertilization. *Soil Till. Res.* **91**, 120–130 (2006).

405 Weil, R.R. & Samaranayake, A. Effects of winged bean on a following maize crop. *Exp. Agric.* **27**, 329-338 (1991).

406 Wesley, R.A., Elmore, C.D. & Spurlock, S.R. Deep tillage and crop rotation effects on cotton, soybean, and grain sorghum on clayey soils. *Agron. J.* **93**, 170-178 (2001).

407 West, T.D., Griffith, D.R., Steinhardt, G.C., Kladivko, E.J. & Parsons, S.D. Effect of tillage and rotation on agronomic performance of corn and soybean: twenty-year study on dark silty clay loam soil. *J. Prod. Agric* **9**, 241-248 (1996).

408 Westphal, A. & Scott, A.W. Implementation of soybean in cotton cropping sequences for management of reniform nematode in South Texas. *Crop Sci.* **45**, 233-239 (2005).

409 Whalen, J.K., Hu, Q. & Liu, A. Compost applications increase water-stable aggregates in conventional and no-tillage systems. *Soil Sci. Soc. Am. J.* **67**, 1842-1847 (2003).

Widdowson, F. V., Penny, A., Gutteridge, R. J., Darby, R. J. & Hewitt, M. V. Tests of amounts and times of application of nitrogen and of sequential sprays of aphicide and

fungicides on winter wheat, following either beans or wheat, and the effects of take-all (*Gaeumannomyces graminis var. tritici*), on two varieties at Saxmundham, Suffolk 1980–3. *J. Agric. Sci.* **105**, 97–122 (1985).

Wiedenfeld, R.P. Previous-crop effects on sugarcane responses to nitrogen fertilization. *Agron. J.* **90**, 161-165 (1998).

Wieme, R.A., Carpenter-Boggs, L.A., Crowder, D.W., Murphy, K.M. & Reganold, J.P. Agronomic and economic performance of organic forage, quinoa, and grain crop rotations in the Palouse region of the Pacific Northwest, USA. *Agr. Syst.* **177**, 102709 (2020).

Wilhelm, W.W. & Wortmann, C.S. Tillage and rotation interactions for corn and soybean grain yield as affected by precipitation and air temperature. *Agron. J.* **96**, 425-432 (2004).

Wolfe, A.M., Eckert, D.J. & Eckert, D.J. Crop sequence and surface residue effects on the performance of no-till corn grown on a poorly drained soil. *Agron. J.* **91**, 363-367 (1999).

Wolfe, A.M., Eckert, D.J. & Eckert, D.J. Crop sequence and surface residue effects on the performance of no-till corn grown on a poorly drained soil. *Agron. J.* **91**, 363-367 (2000).

Woźniak, A. & Soroka, M. Effect of crop rotation and tillage system on the weed infestation and yield of spring wheat and on soil properties. *Appl. Ecol. Env. Res.* **16**, 3087-3096 (2018).

Woźniak, A. Effect of cereal monoculture and tillage systems on grain yield and weed infestation of winter durum wheat. *Int. J. Plant Prod.* **14**, 1-8 (2020).

Woźniak, A. Effect of crop rotation and cereal monoculture on the yield and quality of winter wheat grain and on crop infestation with weeds and soil properties. *Int. J. Plant. Prod.* **13**, 177-182 (2019).

Woźniak, A. Effect of various systems of tillage on winter barley yield, weed infestation and soil properties. *Appl Ecol Env Res* **18**, 3483-3496 (2020).

Woźniak, A., Nowak, A., Haliniarz, M. & Gawęda, D. Yield and economic results of spring barley grown in crop rotation and in monoculture. *Pol. J. Environ. Stud.* **28**, 2441-2448 (2019).

Woźniak, A., Wesołowski, M. & Soroka, M. Effect of long-term reduced tillage on grain yield, grain quality and weed infestation of spring wheat. *J. Agric. Sci. Technol.* **17**, 899-908 (2015).

Wright, A.T. Yield effect of pulses on subsequent cereal crops in the Northern prairies. *Can. J. Plant Sci.***70**, 1023-1032 (1990).

Yadav, R.L., Singh, V.K., Dwivedi, B.S. & Shukla, A.K. Wheat productivity and N use-efficiency as influenced by inclusion of cowpea as a grain legume in a rice–wheat system. *J. Agric. Sci.* **141**, 213-220 (2003).

Yau, S. K., Bounejmate, M., Ryan, J. & Nassar, A. Sustainable barley-legume rotations for semi-arid areas of Lebanon. *Challenges Strateg. Dryl. Agric.* **32**, 219–227 (2004).

Yusuf, A.A., Abaidoo, R.C., Iwuafor, E.N.O., Olufajo, O.O. & Sanginga, N. Rotation effects of grain legumes and fallow on maize yield, microbial biomass and chemical properties of an Alfisol in the Nigerian savanna. *Agric. Ecosyst. Environ.* **129**, 325-331 (2009).

Yusuf, A.A., Iwuafor, E.N.O., Abaidoo, R.C., Olufajo, O.O. & Sanginga, N. Grain legume rotation benefits to maize in the Northern Guinea savanna of Nigeria: Fixed-nitrogen versus other rotation effects. *Nutr. Cycl. Agroecosystems* **84**, 129-139 (2009).

Zebarth, B.J. & Sheard, R.W. Yield and protein response of hard red winter wheat to rate of
427 nitrogen fertilization and previous legume crop. *Can. J. Plant Sci.***72**, 21-25 (1992).

Zentner, R.P. et al. Long-term assessment of management of an annual legume green manure
428 crop for fallow replacement in the brown soil zone. *Can. J. Plant Sci.* **84**, 11-22 (2004).

Zentner, R.P., Campbell, C.A., Bowren, K.E. & Edwards, W. Effects of crop rotations and
429 fertilization on yields and quality of spring wheat grown on a Black Chernozem in
Northcentral Saskatchewan. *Can. J. Plant Sci.* **70**, 383-397 (1990).

Zentner, R.P., Campbell, C.A., Selles, F., Jefferson, P.G. & Lemke, R. Effect of fallow
430 frequency, flexible rotations, legume green manure, and wheat class on the economics of
wheat production in the Brown soil zone. *Can. J. Plant Sci.* **86**, 413-423 (2006).

Zhang, B., Drury, C. F., Yang, X., Reynolds, D. & Zhang, X. Crop rotation, red clover and
431 cropping history influences microbial amino sugars of a clay loam soil. *Soil Sci. Soc. Am. J.*
78, 818–824 (2014).

Zhang, X., Bi, J., Sun, H., Zhang, J. & Zhou, S. Greenhouse gas mitigation potential under
432 different rice-crop rotation systems: From site experiment to model evaluation. *Clean
Technol. Environ. Policy* **21**, 1587-1601 (2019).

Zhao, X., Wang, S. & Xing, G. Maintaining rice yield and reducing N pollution by
433 substituting winter legume for wheat in a heavily-fertilized rice-based cropping system of
Southeast China. *Agric. Ecosyst. Environ.* **202**, 79-89 (2015).

Zhong, C., Yang, B.J., Zhang, P., Li, P. & Huang, G.Q. Effect of paddy-upland rotation with
434 different winter corps on rice yield and CH_4 and N_2O emissions in paddy fields. *Acta Agric.
Nucl. Sin.* **33**, 379-388 (2019).

Zhou, G.Y. et al. No-tillage effects on corn and soybean yield and profitability: based on a
435 long-term field trial of black soil in northeast China. *J. Jilin Agri. Univ.* **37**，260-267 (2015).

Zhou, T. et al. Effects of alfalfa and kentucky bluegrass rotations on agronomic characters and
436 nutritional quality. *Acta Agrestia Sin.* **28**, 720-726 (2020).

Zielke, R. C. & Christenson, D. R. Organic carbon and nitrogen changes in soil under selected
437 cropping systems. *Soil Sci. Soc. Am. J.* **50**, 363–367 (1986).

Zingore, S., Murwira, H.K., Delve, R.J. & Giller, K.E. Variable grain legume yields,
438 responses to phosphorus and rotational effects on maize across soil fertility gradients on
African smallholder farms. *Nutr. Cycl. Agroecosystems* **80**, 1-18 (2008).

439 Adhikary, S., Bagchi, D. K., Ghosal, P., Banerjee, R. N. & Chatterjee, B. N. Studies on
maize‐legume intercropping and their residual effects on soil fertility status and succeeding
crop in upland situation. *Journal of Agronomy and Crop Science-Zeitschrift Fur Acker Und
Pflanzenbau* **167**, 289-293, doi:10.1111/j.1439-037X.1991.tb00959.x (1991).

Bado, B. V., Bationo, A. & Cescas, M. P. Assessment of cowpea and groundnut contributions
to soil fertility and succeeding sorghum yields in the Guinean savannah zone of Burkina Faso
440 (West Africa). *Biology and Fertility of Soils* **43**, 171-176, doi:10.1007/s00374-006-0076-7
(2006).

Bationo, A. & Ntare, B. R. Rotation and nitrogen fertilizer effects on pearl millet, cowpea and
groundnut yield and soil chemical properties in a sandy soil in the semi-arid tropics, West

Africa. *Journal of Agricultural Science* **134**, 277-284, doi:10.1017/s0021859699007650 (2000).

Do Thi, X. *et al.* Different crop rotation systems as drivers of change in soil bacterial community structure and yield of rice, Oryza sativa. *Biology and Fertility of Soils* **48**, 217-225, doi:10.1007/s00374-011-0618-5 (2012).

Feng, B. *et al.* Effects of crop rotation systems on microbial structure under low N application in rice field. *Journal of Plant Nutrition* **43**, 500-511, doi:10.1080/01904167.2019.1685104 (2020).

Grabau, Z. J. & Chen, S. Determining the role of plant-parasitic nematodes in the corn-soybean crop rotation yield effect using nematicide application: I. Corn. *Agronomy Journal* **108**, 782793, doi:10.2134/agronj2015.0431 (2016).

Izaurralde, R. C., Choudhary, M., Juma, N. G., McGill, W. B. & Haderlein, L. Crop and nitrogen yield in legume-based rotations practiced with zero tillage and low-input methods. *Agronomy Journal* **87**, 958-964, doi:10.2134/agronj1995.00021962008700050031x (1995).

Stecker, J. A., Buchholz, D. D., Hanson, R. G., Wollenhaupt, N. C. & McVay, K. A. Tillage and rotation effects on corn yield response to fertilizer nitrogen on Aqualf soils. *Agronomy Journal* **87**, 409-415, doi:10.2134/agronj1995.00021962008700030004x (1995).

Ryan, J., Singh, M. & Christiansen, S. Assessment of long-term barley-legume rotations in a typical Mediterranean agro-ecosystem: grain and straw yields. *Archives of Agronomy and Soil Science* **58**, 233-246, doi:10.1080/03650340.2010.514267 (2012).

Liu, D. L., Chan, K. Y. & Conyers, M. K. Simulation of soil organic carbon under different tillage and stubble management practices using the Rothamsted carbon model. *Soil & Tillage Research* **104**, 65-73, doi:10.1016/j.still.2008.12.011 (2009).

Badaruddin, M. & Meyer, D. W. Forage legume effects on soil nitrogen and grain yield, and nitrogen nutrition of wheat. *Agronomy Journal* **81**, 419-424, doi:10.2134/agronj1989.00021962008100030004x (1989).

Kermah, M. *et al.* Legume-maize rotation or relay? Options for ecological intensification of smallholder farms in the Guinea savanna of northern Ghana. *Experimental Agriculture* **55**, 673-691, doi:10.1017/s0014479718000273 (2019).

Kazula, M. J., Lauer, J. G. & Arriaga, F. J. Crop rotation effect on selected physical and chemical properties of Wisconsin soils. *Journal of Soil and Water Conservation* **72**, 553-563, doi:10.2489/jswc.72.6.553 (2017).

Mason, M. G. & Rowland, I. C. Nitrogen fertiliser response of wheat in lupin-wheat, subterranean clover-wheat and continuous wheat rotations. *Australian Journal of Experimental Agriculture* **30**, 231-236, doi:10.1071/ea9900231 (1990).

Muchane, M. N. *et al.* Influence of improved fallow systems and phosphorus application on arbuscular mycorrhizal fungi symbiosis in maize grown in western Kenya. *Agroforestry Systems* **78**, 139-150, doi:10.1007/s10457-009-9249-3 (2010).

Muleba, N. Effects of cowpea, crotalaria and sorghum crops and phosphorus fertilizers on maize productivity in semi-arid West Africa. *Journal of Agricultural Science* **132**, 61-70, doi:10.1017/s0021859698006182 (1999).

Samake, O., Stomph, T. J., Kropff, M. J. & Smaling, E. M. A. Integrated pearl millet management in the sahel: Effects of legume rotation and fallow management on productivity and Striga hermonthica infestation. *Plant and Soil* **286**, 245-257, doi:10.1007/s11104-0069041-3 (2006).

Yadav, R. L. & Verma, R. P. Crop residue management to conserve soil organic matter content in sugarcane-based crop rotations. *Bioresource Technology* **51**, 241-245, doi:10.1016/09608524(94)00135-n (1995).

Rao, A. V., Tarafdar, J. C., Sharma, S. K., Praveenkumar & Aggarwal, R. K. Influence of cropping systems on soil biochemical properties in an arid rain-fed environment. *Journal of Arid Environments* **31**, 237-244, doi:10.1006/jare.1995.0063 (1995).

Tarawali, S. A. & Peters, M. The potential contribution of selected forage legume pastures to cereal production in crop-livestock farming systems. *Journal of Agricultural Science* **127**, 175182, doi:10.1017/s0021859600077959 (1996).

Jagadamma, S., Lal, R., Hoeft, R. G., Naffiger, E. D. & Adee, E. A. Nitrogen fertilization and cropping system impacts on soil properties and their relationship to crop yield in the central Corn Belt, USA. *Soil & Tillage Research* **98**, 120-129, doi:10.1016/j.still.2007.10.008 (2008).

Stevenson, F. C. & vanKessel, C. The nitrogen and non-nitrogen rotation benefits of pea to succeeding crops. *Canadian Journal of Plant Science* **76**, 735-745, doi:10.4141/cjps96-126 (1996).

Sun, B. *et al.* No tillage combined with crop rotation improves soil microbial community composition and metabolic activity. *Environmental Science and Pollution Research* **23**, 64726482, doi:10.1007/s11356-015-5812-9 (2016).

Riedell, W. E., Pikul, J. L., Jr., Jaradat, A. A. & Schumacher, T. E. Crop rotation and nitrogen input effects on soil fertility, maize mineral nutrition, yield, and seed composition. *Agronomy Journal* **101**, 870-879, doi:10.2134/agronj2008.0186x (2009).

Yadav, R. L. & Prasad, S. R. Maximizing sugarcane yield by increasing plant population density, minimizing NO3-N leaching and improving soil organic matter in different crop rotations. *Journal of Agronomy and Crop Science-Zeitschrift Fur Acker Und Pflanzenbau* **178**, 117-123, doi:10.1111/j.1439-037X.1997.tb00359.x (1997).

Cui, H. *et al.* Effect of crop rotation and irrigation on wheat yield and water-use efficiency. *Chinese Journal of Eco-Agriculture* **17**, 479-483, doi:10.3724/sp.j.1011.2009.00479 (2009).

Fang, Y., Wang, F., Li, Q., Lin, C. & He, C. Effect of continuous paddy-upland crop rotation on bacterial community structure in cold waterlogged paddy soil. *Acta Pedologica Sinica* **55**, 515-525 (2018).

Li, Q. *et al.* Effects of paddy-upland rotation on the nutrient activation of soil carbon, nitrogen and phosphorus in cold waterlogged paddy field. *Journal of Soil and Water Conservation* **29**, 113-117 (2015).

Xu, W. *et al.* Effects of crop stubbles on cotton yield and soil environment in continuously cropped cotton field. *Transactions of the Chinese Society of Agricultural Engineering* **27**, 271275 (2011).

Zhang, X. *et al.* Yield and profitability of corn and soybean under different tillage in the black

soils of Northeast China. *Chinese Journal of Eco-Agriculture* **16**, 858-864, doi:10.3724/sp.j.1011.2008.00858 (2008).

Xu, H. Test on the cultivation model of broad bean and highland barley reduce fertilizer and increasing efficiency. *Sci. Tech. Qinghai Agric. For.*, 79-81 (2018).

Dong, C.H. Studies on reddish paddy field microbial properties of long-term fertilization and crop rotation system[D]. Hunan Agricultural University, 2014.

Gu, J.L. Effects of different planting modes on soil characters and maize growth and development and yield[D]. Northeast Agricultural University, 2019.

Guo, J.R. Impacts of long-term different fertilizer cropping regimes on maize yield and soil quality, and greenhouse gas emission effects on blacks soil region of Northeast China [D]. Nanjing Agricultural University, 2015.

Cui,X.L. Studies on the effects of previous crops on yield and qulity of tobacco[D]. Hunan Agricultural University, 2009.

Li, H.Y. Eevaluation of nutrient utilization efficiency and soil fertility quality in different planting patterns of double-cropping region in southern part of north china plain[D]. Henan Normal University, 2018.

Li, W.B. Effects of different double-cropping-rice-based rotations on rice yield and nutrient runoff loss in paddy field [D]. Hunan Agricultural University, 2018.

Liang, Z.T. Characteristics of soil microbial communities and soil nutrients under different forage-crop rotations in the longdong loess plateau[D]. Lanzhou University, 2018.
