# Supplementary material for: Global systematic review with meta-analysis reveals yield advantage of legume-based rotations and its drivers
Source: Nat Commun. 2022 Aug 22;13:4926. doi: 10.1038/s41467-022-32464-0 (PMC9395539; doi:10.1038/s41467-022-32464-0)
Supplement: Supplementary file 2 — Reporting Summary [file 41467_2022_32464_MOESM2_ESM.pdf]

nature portfolio

Corresponding author(s): Huadong Zang

Last updated by author(s): Jul 12, 2022

# Reporting Summary

Nature Portfolio wishes to improve the reproducibility of the work that we publish. This form provides structure for consistency and transparency in reporting. For further information on Nature Portfolio policies, see our Editorial Policies and the Editorial Policy Checklist.

## Statistics

For all statistical analyses, confirm that the following items are present in the figure legend, table legend, main text, or Methods section.

| n/a | Confirmed | |
|---|---|---|
| ☐ | ☒ | The exact sample size ($n$) for each experimental group/condition, given as a discrete number and unit of measurement |
| ☐ | ☒ | A statement on whether measurements were taken from distinct samples or whether the same sample was measured repeatedly |
| ☐ | ☒ | The statistical test(s) used AND whether they are one- or two-sided<br>*Only common tests should be described solely by name; describe more complex techniques in the Methods section.* |
| ☐ | ☒ | A description of all covariates tested |
| ☐ | ☒ | A description of any assumptions or corrections, such as tests of normality and adjustment for multiple comparisons |
| ☐ | ☒ | A full description of the statistical parameters including central tendency (e.g. means) or other basic estimates (e.g. regression coefficient) AND variation (e.g. standard deviation) or associated estimates of uncertainty (e.g. confidence intervals) |
| ☐ | ☒ | For null hypothesis testing, the test statistic (e.g. $F$, $t$, $r$) with confidence intervals, effect sizes, degrees of freedom and $P$ value noted<br>*Give P values as exact values whenever suitable.* |
| ☒ | ☐ | For Bayesian analysis, information on the choice of priors and Markov chain Monte Carlo settings |
| ☐ | ☒ | For hierarchical and complex designs, identification of the appropriate level for tests and full reporting of outcomes |
| ☐ | ☒ | Estimates of effect sizes (e.g. Cohen's $d$, Pearson's $r$), indicating how they were calculated |

*Our web collection on statistics for biologists contains articles on many of the points above.*

## Software and code

Policy information about availability of computer code

| | |
|---|---|
| Data collection | Peer-reviewed publications that published prior to October 2020, which investigated the effects of legume pre-crop on main crop yield were collected by searching the ISI. Web of Science, Google Scholar, and the China National Knowledge Infrastructure. GetData Graph Digitizer version 2.26 (http://getdatagraphdigitizer.com/) was used to extracted data from the figures. |
| Data analysis | The data analysis was conducted in R4.0.3 (R Core Team, 2021), using packages including 'lme4 (version 1.1-27.1)', 'metaforest (version 0.1.3)'. Details were reported in statistical analysis section of the Methods. The R scripts needed to reproduce the analysis have been deposited in Figshare (10.6084/m9.figshare.20290959). |

For manuscripts utilizing custom algorithms or software that are central to the research but not yet described in published literature, software must be made available to editors and reviewers. We strongly encourage code deposition in a community repository (e.g. GitHub). See the Nature Portfolio guidelines for submitting code & software for further information.

## Data

Policy information about availability of data

All manuscripts must include a data availability statement. This statement should provide the following information, where applicable:

- Accession codes, unique identifiers, or web links for publicly available datasets
- A description of any restrictions on data availability
- For clinical datasets or third party data, please ensure that the statement adheres to our policy

The datasets generated during and/or analyzed during the current study have been deposited in Figshare (10.6084/m9.figshare.20290923)



March 2021

# Field-specific reporting

Please select the one below that is the best fit for your research. If you are not sure, read the appropriate sections before making your selection.

☐ Life sciences ☐ Behavioural & social sciences ☒ Ecological, evolutionary & environmental sciences

For a reference copy of the document with all sections, see nature.com/documents/nr-reporting-summary-flat.pdf

# Ecological, evolutionary & environmental sciences study design

All studies must disclose on these points even when the disclosure is negative.

**Study description**

We conducted a global meta-analysis with 11,768 paired observations of legume-based and non-legume cropping systems from 462 studies, to evaluate the responses of main crop yield to legume-based rotation. We identify the most important factors ("moderators") of the legume pre-crop effect on main crop yield in the dataset based on random-forest meta-analysis. We design a linear mixed-effects meta-regression with selected moderators, including "study" as a random factor, and use this statistical model to quantify the legume pre-crop effect on main crop yield globally.

**Research sample**

The dataset was built by collecting literature data from Web of Science, Google Scholar, and the China National Knowledge Infrastructure. The dataset was obtained from 476 peer-reviewed publications after strict screening of 16630 papers. The dataset includes sufficient information for our analysis, such as the yields of main crops grown in legume-based and non-legume cropping systems, the soil parameters, the climate variables, and management practices. The 476 publications were listed in Supplementary Note in Supplementary Materials.

**Sampling strategy**

We systematically searched all peer-reviewed publications that published prior to October 2020, which investigated the effects of legume pre-crop on main crop yield using the Web of Science (Core Collection; http://www.webofknowledge.com), Google Scholar (http://scholar.google.com), and the China National Knowledge Infrastructure (CNKI; https://www.cnki.net) with the search term: "("crop* rotation*" OR "crop* sequence*" OR "sequential crop*" OR "successive crop*" OR "ley farming" OR "sequence* of plant species" OR "sequence* of crops") AND ("yield*")", and also searched for references within these papers. We also checked the reference list within the previously published rotation effect meta-analyses and reviews. Publications were screened based on the following criteria: (1) the rotation experiment was conducted under field conditions and contained side-by-side comparisons of legume and non-legume pre-crop rotations with the same main crop; (2) subsequent crop yield data were reported or could be calculated; (3) the initial climatic conditions, soil properties, and main crop management practices were the same; (4) location of the experiment was stated.

**Data collection**

Jie Zhao and Yadong Yang collected the data. For each study, we extracted the means, the number of replications, and standard deviations of the main crop yield, if reported. When an original study reported the results graphically, we used GetData Graph Digitizer (http://getdatagraphdigitizer.com/) to extract data from the figures. We also extracted latitude, longitude, altitude, mean annual temperature (MAT, °C), mean annual precipitation (MAP, mm), annual aridity index, soil pH in water, soil carbon content (SOC, g kg-1), soil total N concentration (STN, g kg-1), soil texture, preceding legume crop species, legume crop purpose, main crop species, crop types, number of crops per year, crop diversity, rotation cycle, nitrogen fertilizer rate (kg ha-1), irrigation, tillage, and residue management from original or cited papers, or cited data sources. MAT (if NA), MAP(if NA), and annual aridity index were retrieved from the WorldClim database (WorldCli v 2.1, https://worldclim.org/data/worldclim21.html) using the location information. The annual aridity index was calculated as the ratio of mean annual precipitation to mean annual potential evapotranspiration. Soil pH, SOC, STN, and soil texture (if NA) were retrieved from the HWSD database (HWSD v 1.2, http://www.fao.org/soils-portal/data-hub/soil-maps-and-databases/harmonized-world-soil-database-v12/en/) using the location information. Soil texture was classified into 11 textural classes and further grouped into three categories (coarse, medium, and fine) based on the USDA textural classes of soils. Legume purpose was classified into three groups (grain, fodder, and green manure). Crop diversity is defined as the number of crop species × number of crop functional groups × number of crop species per year. Rotation cycle is the number of repetitions of a cropping sequence. Management practices including residue management, conservation tillage, irrigation practices, N fertilizer rate, and rotation cycle were recorded for each study as categorical or continuous variables where possible. Residue management was treated as a binary variable (retained/removed), where 'retained' indicates that crop residues were retained in the field following harvest, and 'removed' indicates that residues were physically removed from the field or burned following harvest. Conservation tillage was also treated as a binary variable (yes/no), where 'yes' indicated that the main crop was tilled by conservation tillage including no-till, strip-till, ridge-till, and 'no' indicated conventional tillage including mold board and chisel plow applied to the main crop. Irrigation practices (yes/no) were recorded when available, with cells left blank when irrigation practices were unclear.

**Timing and spatial scale**

The searching for publication and data collection continuously proceeded from June 6th, 2018 to April 6th, 2022.
The timing scale of the observations in original studies ranged from 1959 to 2020.
The spatial scale of the observations were distributed in global farmland ecosystems.

**Data exclusions**

To better represent responses of legume-based rotation effect under field conditions, we did not include greenhouse, pot, and micro-plot studies.

**Reproducibility**

The study is fully reproducible using the data and methods detailed in the manuscript.

**Randomization**

The data were analyzed with a mixed effect model. Bootstrapping sampling was used. Grouping was based on a machine-learning model selection approach.

**Blinding**

Complete blinding in bootstrapping process.

Did the study involve field work? ☐ Yes ☒ No

# Reporting for specific materials, systems and methods

We require information from authors about some types of materials, experimental systems and methods used in many studies. Here, indicate whether each material, system or method listed is relevant to your study. If you are not sure if a list item applies to your research, read the appropriate section before selecting a response.

## Materials & experimental systems

| n/a | Involved in the study |
| --- | --- |
| ☒ | ☐ Antibodies |
| ☒ | ☐ Eukaryotic cell lines |
| ☒ | ☐ Palaeontology and archaeology |
| ☒ | ☐ Animals and other organisms |
| ☒ | ☐ Human research participants |
| ☒ | ☐ Clinical data |
| ☒ | ☐ Dual use research of concern |

## Methods

| n/a | Involved in the study |
| --- | --- |
| ☒ | ☐ ChIP-seq |
| ☒ | ☐ Flow cytometry |
| ☒ | ☐ MRI-based neuroimaging |
