# Supplementary material for: Global systematic review with meta-analysis reveals yield advantage of legume-based rotations and its drivers
Source: Nat Commun. 2022 Aug 22;13:4926. doi: 10.1038/s41467-022-32464-0 (PMC9395539; doi:10.1038/s41467-022-32464-0)
Supplement: Supplementary file 3 — Description of Additional Supplementary Files [file 41467_2022_32464_MOESM3_ESM.pdf]

Title: Supplementary data 1
Description: Publications used for the meta-analysis
